# Solvent-Dependent Phytochemical Profiles, Antioxidant and Antimicrobial Bioactivities, and Melanoma-Selective Cytotoxicity of *Inula helenium* L. Root Extracts

**DOI:** 10.3390/molecules31173019

**Published:** 2026-08-28

**Authors:** Asta Mažeikienė, Miglė Kapliukaitė, Agnė Kirkliauskienė, Žygimantas Stasaitis, Simonas Mažeika, Neringa Burokienė, Aušra Repečkienė

**Affiliations:** 1 Department of Physiology, Biochemistry, Microbiology and Laboratory Medicine, Institute of Biomedical Sciences, Faculty of Medicine, Vilnius University, M. K. Čiurlionio St. 21, LT-03101 Vilnius, Lithuaniaagne.kirkliauskiene@mf.vu.lt (A.K.);; 2 Clinics of Internal Diseases and Family Medicine, Institute of Clinical Medicine, Faculty of Medicine, Vilnius University, M. K. Čiurlionio St. 21, LT-03101 Vilnius, Lithuania; neringa.burokiene@mf.vu.lt; 3 Pharmacy and Pharmacology Center, Institute of Biomedical Sciences, Faculty of Medicine, Vilnius University, M. K. Čiurlionio St. 21, LT-03101 Vilnius, Lithuania; ausra.repeckiene@mf.vu.lt

**Keywords:** solvent polarity, raw material origin, UV–Vis fingerprinting, multivariate analysis, bioactivity profiling, SK-MEL-28

## Abstract

*Inula helenium* L. (elecampane) is a traditional medicinal plant whose roots contain both hydrophilic polyphenols and lipophilic sesquiterpene lactones. However, the influence of the raw material origin and the extraction solvent on its combined bioactivity profile remains unclear. Moreover, although isolated sesquiterpene lactones from *I. helenium* have been extensively investigated for their anticancer properties, the bioactivity of whole root extracts against human melanoma cells has remained largely underexplored. This study aimed to systematically compare the phytochemical characteristics and biological activity of *I. helenium* root extracts obtained from three distinct raw material sources and three solvents of increasing polarity, and to identify the main factors associated with the observed bioactivity through multivariate integration. In this study, *I. helenium* roots from three distinct sources—commercially available dried roots, wild-collected roots, and cultivated roots collected from the Botanical Garden of Vilnius University—were extracted with three solvents of increasing polarity (*n*-hexane, ethyl acetate, and 70% ethanol). Each extract was characterized by total phenolic content, four antioxidant assays (DPPH, ABTS, FRAP, CUPRAC), UV–Vis fingerprinting, antimicrobial testing against *Staphylococcus aureus, Klebsiella pneumoniae*, and *Candida albicans*, and a CCK-8 assay on SK-MEL-28 human melanoma and HEK293 non-cancerous cells. Multivariate integration (PCA, HCA, Spearman rank correlation) identified solvent polarity as the dominant driver of both the phytochemical and the biological profile (PC1 and PC2 together explained 87.9% of the total variance). Ethanolic extracts combined the highest phenolic content (up to 38.6 mg GAE/g dry extract) and the strongest antioxidant capacity across all four assays, with maximum values of 177.3 (DPPH), 328.9 (ABTS), 174.1 (FRAP), and 541.6 (CUPRAC) µmol TE/g DE (*p* ≤ 0.004). In the preliminary antimicrobial screening, ethanolic extracts showed near-complete to complete inhibition of *S. aureus* and *C. albicans* at 25 mg/mL, with MIC_90_ values as low as 12.5 mg/mL against *S. aureus* and 6.25 mg/mL against *C. albicans*, whereas *K. pneumoniae* showed only marginal growth inhibition (median values below 20% across the entire tested concentration range). Strikingly, *n*-hexane and ethyl acetate extracts—despite negligible antioxidant activity—produced concentration-dependent cytotoxic effects. Six of the nine extracts reduced SK-MEL-28 viability while sparing HEK293 cells, with the cultivated Botanical Garden *n*-hexane extract showing the greatest difference across the tested cell models (SI > 1.50, *p* = 0.0003). Targeted solvent selection thus enables a single plant material to yield two functionally distinct bioactive fractions: more polar extracts with pronounced antioxidant and antimicrobial activities and less polar extracts with preferential activity against SK-MEL-28 within the cell models tested.

## 1. Introduction

Oxidative stress underlies the pathogenesis of aging and of most non-communicable diseases, including cardiovascular disorders, neurodegeneration, and cancer [1]. Plant-derived phytochemicals have therefore emerged as a promising line of nutraceutical and phytotherapeutic intervention against ROS-mediated damage [2]. In parallel, the accelerating spread of antimicrobial resistance—dominated by WHO-priority pathogens such as carbapenem-resistant *Klebsiella pneumoniae* [3]—has renewed the urgency of identifying plant-based antimicrobial and cytotoxic principles with a favorable safety profile.

*Inula helenium* L., commonly known as elecampane, is a long-standing medicinal plant traditionally used in Europe and Asia for the treatment of respiratory and infectious conditions [4]. It belongs to the genus *Inula* (Asteraceae/Compositae), which comprises fewer than 100 perennial herbaceous species distributed across warm and temperate regions of Asia, Africa and Europe [5]. The species is native to south-eastern Europe and western Asia and is now naturalised across temperate regions of Europe and North America; its thick, fleshy roots and rhizomes, known as Inulae Radix (IR), constitute the principal medicinal plant material. In addition to its use in European folk medicine for chronic cough, bronchitis and dermal ailments [4], IR is also applied in Traditional Chinese Medicine to gastrointestinal disorders and inflammatory conditions, and is officially monographed in European pharmacopoeias for its diuretic, diaphoretic, expectorant and anthelmintic properties [6].

*I. helenium* roots are rich in bioactive constituents—including lipophilic eudesmanolide-type sesquiterpene lactones, phenolic acids, flavonoids, essential oils composed of monoterpenes and sesquiterpenoids, and inulin-type fructans [5]—complemented by water-soluble constituents such as B-group vitamins and vitamin C [7]. This chemical diversity underlies a broad pharmacological potential, ranging from well-established antioxidant and anti-inflammatory activities linked primarily to alantolactone and related sesquiterpene lactones [8] to more recently reported neuroprotective, hepatoprotective, metabolic, and immunostimulatory effects [6,7], keeping the species a subject of active investigation.

Sesquiterpene lactones—most notably alantolactone and isoalantolactone—are the principal bioactive markers of the species [5,9,10]. These lipophilic α,β-unsaturated lactones display a broad pharmacological spectrum, from antimicrobial and anti-inflammatory effects—the latter mediated through modulation of NF-κB and MAPK signalling pathways [9]—to selective cytotoxicity against tumor cells [9,11] with sesquiterpene-lactone-enriched *I. helenium* fractions also shown to potentiate immune-checkpoint-based antitumor therapy in preclinical cancer models [12]. Hydroalcoholic root extracts, in turn, deliver substantial amounts of polyphenolic compounds with measurable antioxidant capacity [13]—indicating that different classes of *I. helenium* metabolites are likely to drive different components of the pharmacological profile.

The antioxidant potential of *Inula* species is closely linked to their polyphenolic content, with chlorogenic acid and other caffeoylquinic acid derivatives repeatedly identified as the principal contributors to radical-scavenging activity in hydroalcoholic extracts of the genus [14]. Because these polyphenolic constituents can act through distinct chemical mechanisms—single-electron transfer, hydrogen-atom transfer, and metal-ion reduction—reliable characterisation of the antioxidant profile of a complex plant matrix requires the parallel use of complementary assays rather than a single measurement, as recently emphasised for polyphenol-rich matrices [15]. In this context, combining radical-scavenging assays (DPPH, ABTS) with reducing-power assays (FRAP, CUPRAC) provides a broader and mechanistically more informative picture of the antioxidant capacity of *I. helenium* extracts prepared with solvents of different polarity.

The antimicrobial spectrum of *I. helenium* has attracted increasing attention in the context of rising antimicrobial resistance. Sesquiterpene lactones from *I. helenium* roots, most notably alantolactone and isoalantolactone, exhibit pronounced antibacterial activity against Gram-positive pathogens, including methicillin-resistant *S. aureus* and other multidrug-resistant strains, together with marked anti-biofilm effects [4,16]. Beyond antibacterial activity, these compounds also display antifungal potential, synergising with conventional antifungal agents such as nystatin against *C. albicans* and lowering the effective doses required for growth inhibition [17]. In contrast, activity against Gram-negative bacteria is generally reported to be markedly weaker, likely reflecting the additional permeability barrier of the outer membrane [18,19].

The cytotoxic potential of *I. helenium* has attracted particular attention as an emerging line of natural-product-based anticancer research. Extracts and isolated sesquiterpene lactones from this species have shown selective cytotoxicity against a broad range of tumor cell lines, including ovarian, cervical, gastric, and pancreatic cancers [9,11]. In contrast, the cytotoxic activity of whole *I. helenium* root extracts against human melanoma cells have, to the best of our knowledge, not yet been investigated, although isolated sesquiterpene lactones (particularly alantolactone and isoalantolactone) from this species have demonstrated anti-melanoma activity [9,20]. Whether this cytotoxic activity is retained in crude root extracts, and how it is influenced by extraction solvent and raw material origin, remains largely underexplored.

Despite extensive research on the pharmacological properties of *I. helenium*, several important questions remain unresolved. First, most published studies rely on a single extraction solvent, preventing direct comparison of phenolic and lipophilic fractions from the same starting material [13,21]. Second, the influence of raw material origin—commercial pharmacy product, cultivated specimen, or wild-collected material—on antioxidant, antimicrobial, and cytotoxic activities has rarely been assessed within a single experimental design. Third, integrated multivariate approaches capable of jointly analysing phytochemical and biological datasets remain scarce for *I. helenium*, despite the increasing use of chemometric methods in natural products research [22].

The present study addresses these gaps by systematically comparing *I. helenium* root extracts obtained from three independent sources—commercially available dried roots (Pharmacy), wild-collected roots (Wild), and cultivated roots collected from the Botanical Garden of Vilnius University (Botanical Garden)—using three solvents of increasing polarity: *n*-hexane, ethyl acetate, and 70% ethanol. All nine origin–solvent combinations were characterized by a multi-method panel comprising total phenolic content, four complementary antioxidant assays (DPPH, ABTS, FRAP, CUPRAC), a UV–Vis phytochemical fingerprint, antimicrobial testing against *Staphylococcus aureus*, *K. pneumoniae*, and *Candida albicans*, and a cytotoxicity assay on the SK-MEL-28 human melanoma and HEK293 non-cancerous cell lines. SK-MEL-28 was selected as a well-characterized human melanoma cell line widely used for evaluating the cytotoxicity of natural products [23]. The eleven bioactivity variables were then integrated by principal component analysis, hierarchical clustering, and Spearman rank correlation to identify the dominant drivers of the phytochemical and biological profile.

## 2. Results

### 2.1. Total Phenolic Content (TPC)

The total phenolic content of *I. helenium* root extracts varied markedly with both extraction solvent and raw material origin, ranging from 7.27 to 38.59 mg GAE/g DE across the nine origin–solvent combinations. Extracts obtained with 70% ethanol consistently exhibited the highest total phenolic content (TPC), whereas ethyl acetate and *n*-hexane extracts produced substantially lower TPC values (7.27–13.37 mg GAE/g DE), irrespective of the raw material source. Among the ethanolic extracts, the wild-collected roots exhibited the highest TPC value (38.59 mg GAE/g DE), exceeding the Pharmacy (13.93 mg GAE/g DE) and Botanical Garden (9.02 mg GAE/g DE) extracts by 2.8- and 4.3-fold, respectively. Solvent had a highly significant global effect on TPC (*p* = 0.004), whereas raw material origin alone was not a significant factor (*p* = 0.399). Complete statistical parameters and median (Q1–Q3) values for all groups are given in Table 1.

### 2.2. Antioxidant Activity

#### 2.2.1. DPPH Radical Scavenging Activity

DPPH radical scavenging activity followed the same solvent-dependent pattern as TPC, with 70% ethanol extracts far exceeding the two less polar solvents. Values ranged from 1.88 µmol TE/g DE (the Botanical Garden ethyl acetate extract) to 177.29 µmol TE/g DE (the Wild 70% ethanol extract)—a nearly 100-fold difference across the nine groups. The Wild 70% ethanol extract exhibited the highest radical-scavenging capacity (177.29 µmol TE/g DE), followed by the Pharmacy (136.24 µmol TE/g DE) and Botanical Garden (94.00 µmol TE/g DE) ethanolic extracts. In contrast, all *n*-hexane and ethyl acetate extracts remained below 10 µmol TE/g DE, indicating very low DPPH radical-scavenging activity in the less polar extracts. The effect of solvent was highly significant (*p* < 0.001), while raw material origin showed a marginally non-significant trend (*p* = 0.078). The only pairwise origin–solvent contrast reaching significance after Bonferroni correction was between the Wild 70% ethanol extract and the Botanical Garden ethyl acetate extract (adjusted *p* = 0.019).

#### 2.2.2. ABTS Radical Scavenging Activity

The ABTS assay revealed the largest absolute Trolox equivalents among the radical-scavenging assays, with values ranging from 7.05 to 328.93 µmol TE/g DE. As in the DPPH assay, 70% ethanol extracts dominated, and the Wild 70% ethanol extract exhibited the highest ABTS radical-scavenging activity. The Wild 70% ethanol extract reached 328.93 µmol TE/g DE—approximately 2.0-fold and 2.6-fold higher than the Pharmacy (162.92 µmol TE/g DE) and Botanical Garden (125.00 µmol TE/g DE) ethanolic extracts, respectively. Notably, the Wild ethyl acetate extract showed appreciable ABTS activity (45.52 µmol TE/g DE), several-fold higher than any other non-ethanolic extract. Solvent was a highly significant factor (*p* = 0.003), while raw material origin showed a marginally non-significant effect (*p* = 0.096). No individual origin–solvent pairwise comparison reached significance after Bonferroni correction.

#### 2.2.3. Cupric Reducing Antioxidant Capacity (CUPRAC)

CUPRAC values were the highest in absolute terms among the four antioxidant assays, ranging from 29.24 to 541.62 µmol TE/g DE. The Wild 70% ethanol extract again exhibited the highest CUPRAC value (541.62 µmol TE/g DE), followed by Pharmacy (451.76 µmol TE/g DE) and Botanical Garden (278.68 µmol TE/g DE) ethanolic extracts. Among the non-ethanolic extracts, the Wild ethyl acetate extract exhibited the highest CUPRAC value (126.11 µmol TE/g DE), exceeding all other non-ethanolic extracts but remaining below the ethanolic extracts. Solvent was a highly significant factor (*p* = 0.002), while raw material origin was not significant (*p* = 0.160). One outlier (the Pharmacy ethyl acetate extract first replicate; TE = 110.39 µmol TE/g DE) was excluded prior to analysis after confirmation by both Grubbs and Dixon’s *Q* tests (*p* < 0.05; see Section 4.10). No individual origin–solvent pairwise comparison reached significance after Bonferroni correction.

#### 2.2.4. Ferric Reducing Antioxidant Power (FRAP)

**Figure 1:**
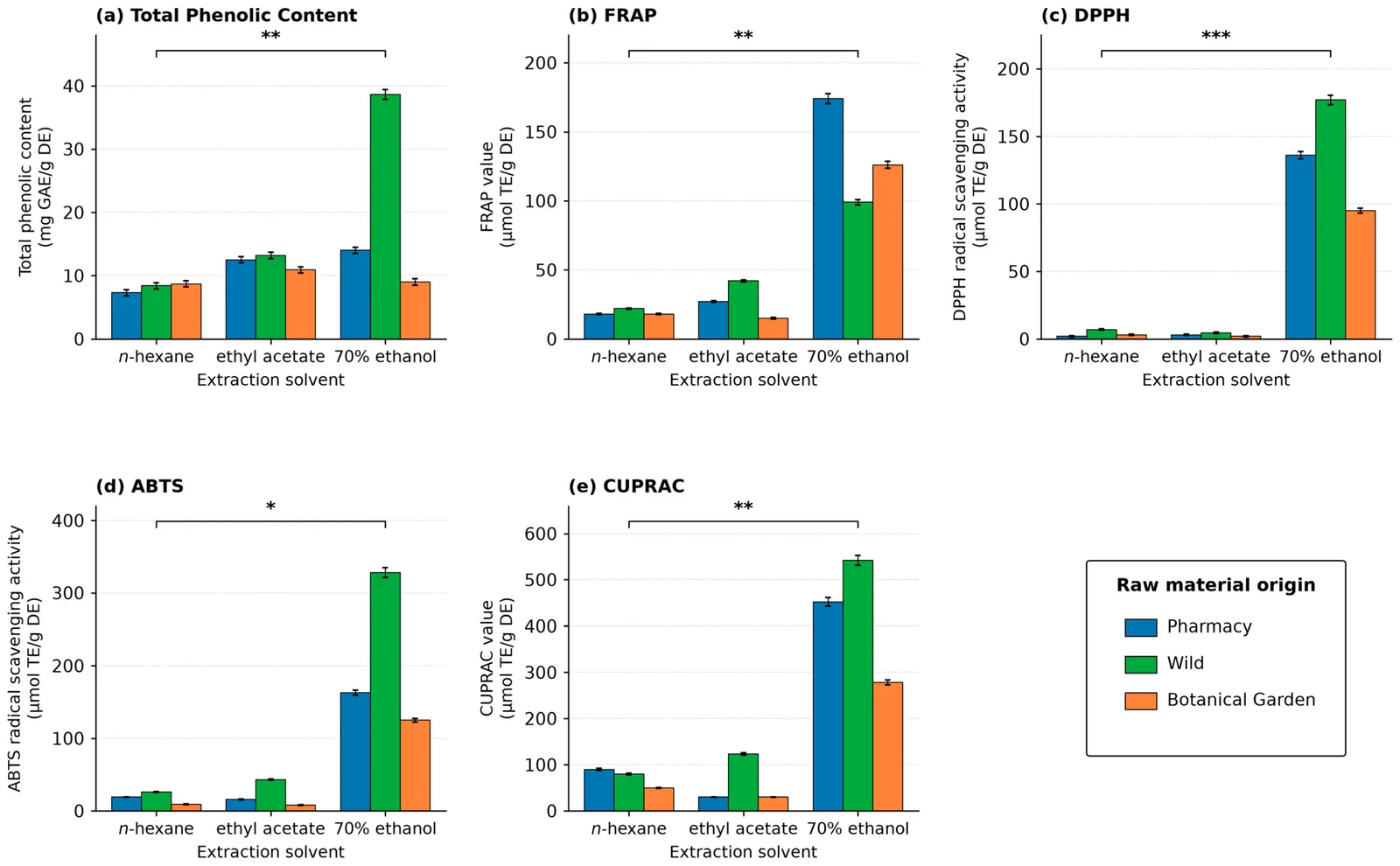
Total phenolic content (TPC) and antioxidant activity of *Inula helenium* L. root extracts: (**a**) TPC (mg GAE/g DE); (**b**) FRAP; (**c**) DPPH; (**d**) ABTS; and (**e**) CUPRAC (all expressed as µmol TE/g DE). Bars represent medians of *n* = 3 biological replicates (each measured in triplicate technical wells); whiskers indicate Q1–Q3. Asterisks denote significant differences between extraction solvents (pooled across raw material origins) from Dunn’s post hoc test with Bonferroni correction following Kruskal–Wallis (* *p* < 0.05, ** *p* < 0.01, *** *p* < 0.001). DE, dry extract; GAE, gallic acid equivalents; TE, Trolox equivalents.

FRAP values ranged from 17.72 to 174.06 µmol TE/g DE, again with 70% ethanol extracts exhibiting the highest FRAP values. However, the FRAP assay was the only one in which the Wild 70% ethanol extract did not exhibit the highest FRAP value. Within the ethanolic series, the reducing capacity followed the order Pharmacy (174.06 µmol TE/g DE) > Botanical Garden (126.32 µmol TE/g DE) > Wild (98.83 µmol TE/g DE)—the inverse of the hierarchy observed in the TPC, DPPH, ABTS, and CUPRAC assays. Among the less polar extracts, the Wild ethyl acetate extract exhibited the highest FRAP value among the non-ethanolic extracts (42.00 µmol TE/g DE). Solvent had a highly significant global effect (*p* = 0.003), while raw material origin alone was not significant (*p* = 0.459). No individual origin–solvent pairwise comparison reached significance after Bonferroni correction. Complete descriptive statistics and pairwise comparisons for all origin–solvent groups are given in Table 1 and Figure 1.

### 2.3. Correlations Between Total Phenolic Content and Antioxidant Activity

**Figure 2:**
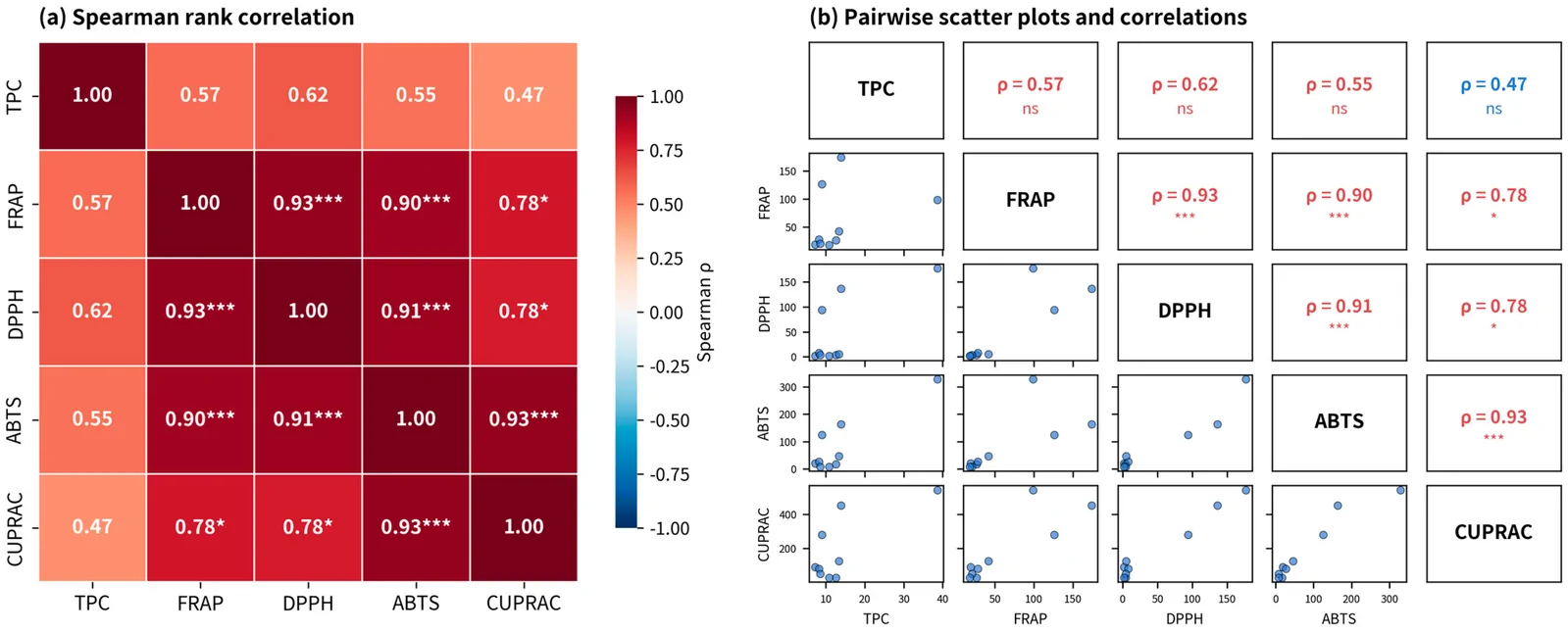
Spearman correlation analysis of total phenolic content (TPC) and four antioxidant activity assays (FRAP, DPPH, ABTS, CUPRAC) of *Inula helenium* L. root extracts (*n* = 9 origin–solvent group medians). (**a**) Heatmap of pairwise Spearman *ρ* values; (**b**) pairwise scatterplot matrix (lower triangle) and *ρ* values (upper triangle). Significance: *** *p* < 0.001, * *p* < 0.05, ns = not significant.

Spearman rank correlation analysis was performed on the median values of the nine origin–solvent groups to evaluate the relationships between total phenolic content and the four antioxidant assays (Figure 2). All four antioxidant assays were strongly and significantly inter-correlated (*ρ* = 0.78–0.93, all *p* < 0.05), with the strongest associations observed between ABTS and CUPRAC (*ρ* = 0.93), DPPH and FRAP (*ρ* = 0.93), and DPPH and ABTS (*ρ* = 0.91). In contrast, the total phenolic content showed only moderate, non-significant correlations with all four antioxidant assays (*ρ* = 0.47–0.62, all *p* > 0.05).

### 2.4. Phytochemical Fingerprinting by UV–Vis Spectroscopy

**Figure 3:**
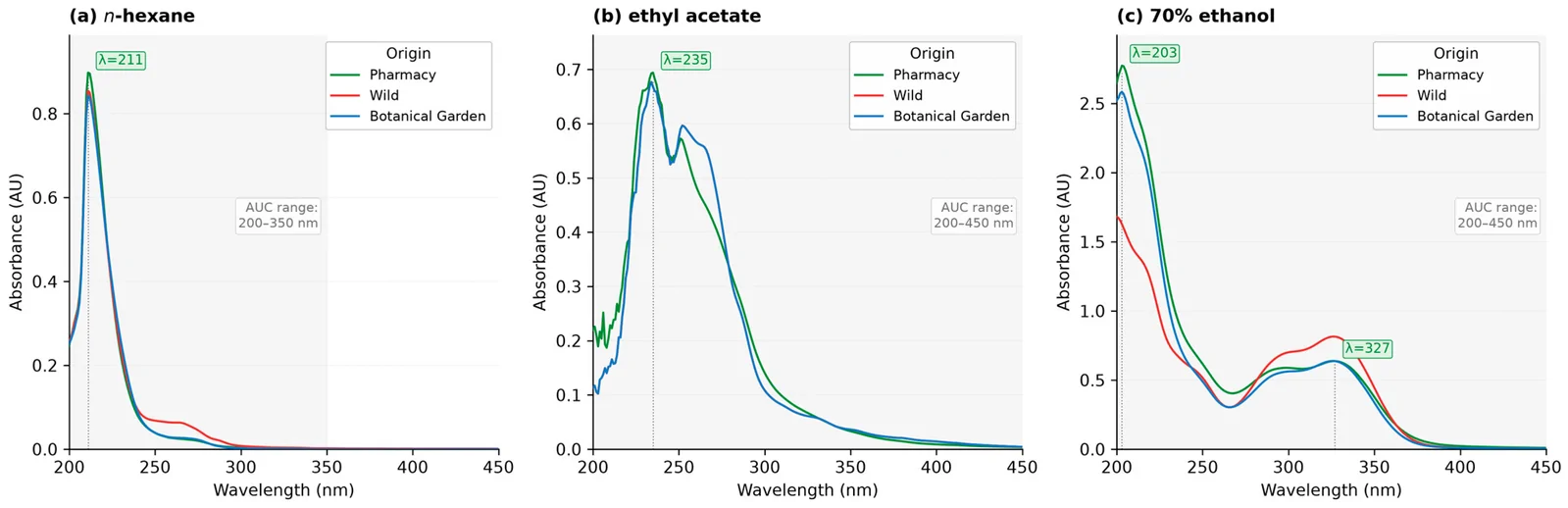
UV–Vis absorbance spectra (200–800 nm) of *Inula helenium* L. root extracts obtained with three solvents of increasing polarity: (**a**) *n*-hexane (0.02 mg/mL); (**b**) ethyl acetate (0.2 mg/mL); (**c**) 70% ethanol (0.2 mg/mL). Each panel shows three spectra corresponding to the three raw material sources: Pharmacy (green), Wild (red) and Botanical Garden (blue). Dotted vertical lines and yellow labels indicate the absorbance maxima (*λ*_max_) detected in the Pharmacy spectrum (*n*-hexane: 211 nm; ethyl acetate: 235 nm; 70% ethanol: 203, 327 nm). Shaded gray regions indicate the wavelength interval used for area-under-the-curve (AUC) integration (200–350 nm for *n*-hexane; 200–450 nm for ethyl acetate and 70% ethanol).

The qualitative phytochemical composition of *I. helenium* root extracts was assessed by UV–Vis spectrophotometry over the 200–800 nm wavelength range, with the most informative absorbance features observed between 200 and 450 nm (Figure 3 and Table 2).

The *n*-hexane extracts displayed a single dominant absorbance maximum at *λ*_max_ = 211 nm (*A* = 0.84–0.90). The corresponding AUC values were low and very similar across raw material sources (18.6–20.0; range 200–350 nm), indicating a highly similar spectral profile irrespective of raw material source (Figure 3a and Table 2). Ethyl acetate extracts produced a more complex profile with two distinct maxima at *λ*_max_ ≈ 234–235 nm and *λ*_max_ ≈ 251–252 nm. The AUC values (44.6–45.8; range 200–450 nm) were almost indistinguishable among the three raw material sources (relative difference < 3%), supporting a highly similar spectral profile among the ethyl acetate extracts (Figure 3b and Table 2). The 70% ethanolic extracts showed two absorbance maxima at *λ*_max_ ≈ 203–210 nm and *λ*_max_ = 326–327 nm and produced by far the highest AUC values (116.8–142.8; range 200–450 nm), exceeding those of the *n*-hexane and ethyl acetate extracts approximately 6- and 3-fold, respectively (Figure 3c and Table 2).

**Figure 4:**
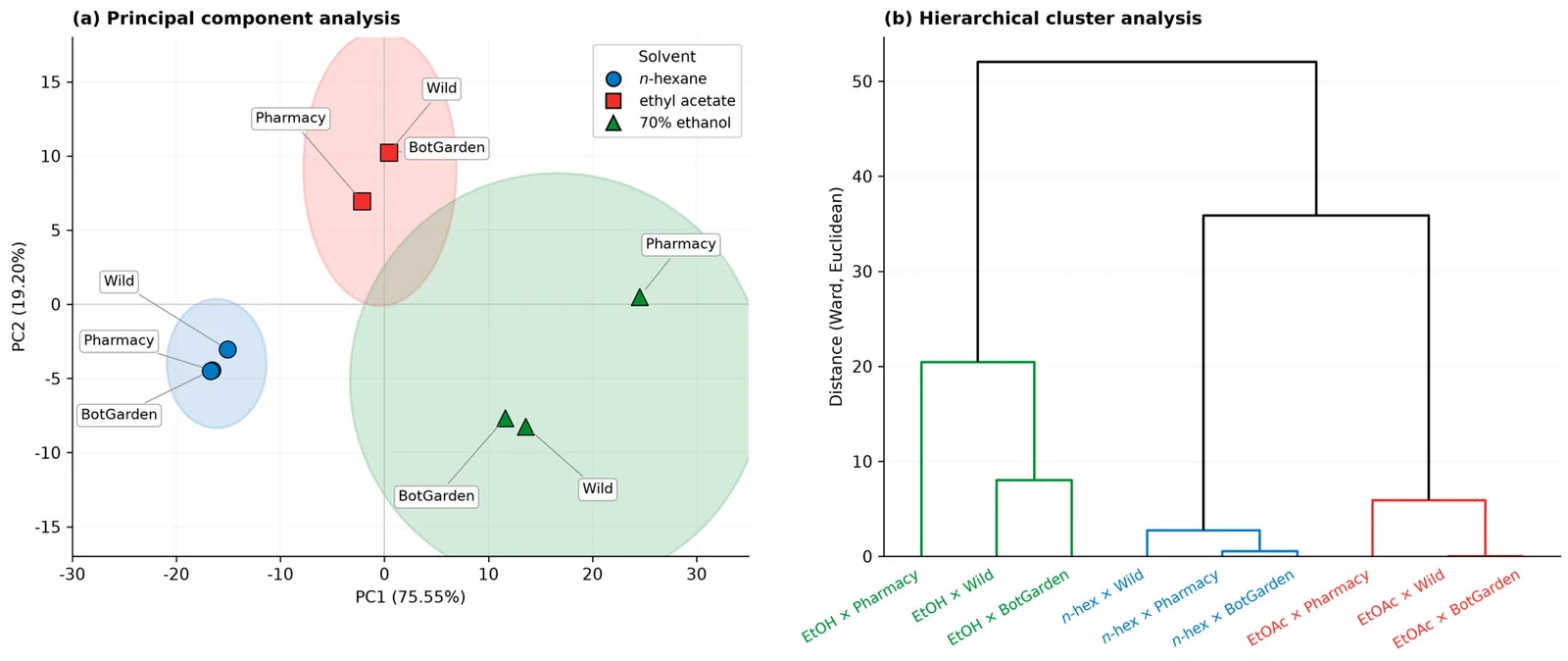
Multivariate analysis of UV–Vis spectra (200–450 nm) of *Inula helenium* L. root extracts. (**a**) Principal component analysis (PCA) score plot of standardized absorbance spectra; PC1 + PC2 explain 94.75% of total variance. Some samples overlap due to spectral similarity (e.g., EtOAc Wild and EtOAc Botanical Garden). (**b**) Hierarchical cluster analysis dendrogram (Ward linkage on Euclidean distances of standardized spectra). Solvent abbreviations used in panel (**b**): *n*-Hex = *n*-hexane; EtOAc = ethyl acetate; EtOH = 70% ethanol. Color coding: blue = *n*-hexane; red = ethyl acetate; green = 70% ethanol.

To objectively assess the contribution of solvent versus raw material source to the spectral profile, the standardized spectra were subjected to principal component analysis (PCA) and hierarchical cluster analysis (HCA) (Figure 4). The first two principal components explained 94.75% of total variance (PC1 = 75.55%, PC2 = 19.20%). All samples were segregated into three distinct clusters according to the extraction solvent—*n*-hexane, ethyl acetate and 70% ethanol—whereas no clear separation was observed according to raw material source, as extracts obtained with the same solvent clustered closely in PC space regardless of raw material source, with the Wild and Botanical Garden ethyl acetate extracts virtually overlapping. The dendrogram derived from Ward linkage on Euclidean distances confirmed the solvent-driven grouping (Figure 4b): a primary split separated the *n*-hexane extracts from a secondary cluster containing the more polar ethyl acetate and 70% ethanol extracts, which further branched into two solvent-specific sub-clusters.

### 2.5. Antimicrobial Activity

**Figure 5:**
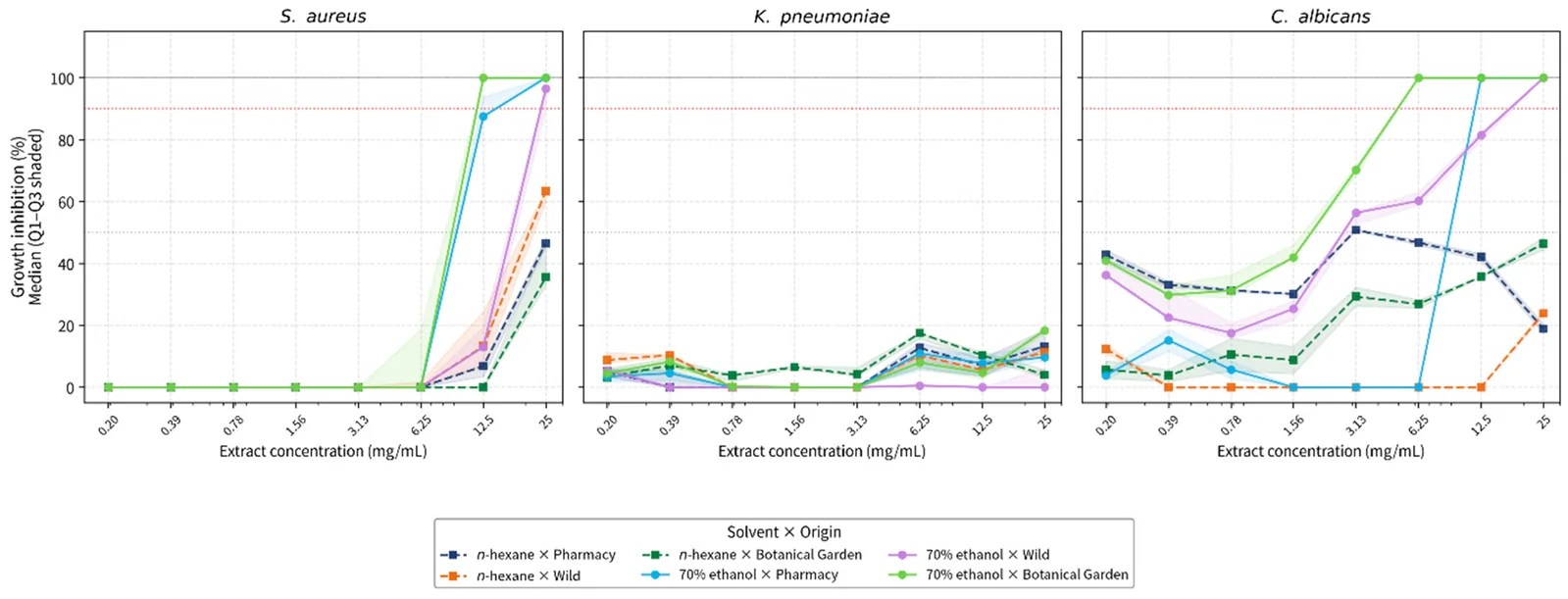
Dose–response profiles of the antimicrobial activity of *Inula helenium* L. root extracts against *Staphylococcus aureus* (Gram-positive), *Klebsiella pneumoniae* (Gram-negative), and *Candida albicans* (yeast). Median growth inhibition (%) is plotted against extract concentration on a logarithmic scale (0.195–25 mg/mL), with the interquartile range (Q1–Q3) shown as a shaded band around each curve. Six experimental conditions are displayed per microorganism: three raw material origins (Pharmacy, Wild, Botanical Garden) combined with two extraction solvents (*n*-hexane, dashed lines with square markers; 70% ethanol, solid lines with circular markers). Reference lines indicate 50% inhibition (gray dotted), 90% inhibition (red dotted), and 100% growth inhibition (solid black). Individual per-well inhibition values were capped at the [0, 100] interval prior to computing medians; a value of 100% therefore denotes complete growth inhibition. MIC_50_ and MIC_90_ values were derived from these profiles using a monotonic dose–response criterion (see Section 4.8) and are reported in Table 3; full median inhibition values at all tested concentrations are provided in Table S1. Ethyl acetate extracts were excluded due to optical interference (see Section 2.5). Data are based on *n* = 3 technical replicate wells per condition within a single independent experiment.

The antimicrobial activity of *I. helenium* root extracts was evaluated against three test microorganisms: the Gram-positive bacterium *S. aureus*, the Gram-negative bacterium *K. pneumoniae*, and the yeast *C. albicans*. Extracts were tested across eight two-fold serial dilutions ranging from 0.195 to 25 mg/mL. Results are summarized in Table 3, with the corresponding dose–response profiles shown in Figure 5; full median inhibition values at all tested concentrations are provided in Table S1. Ethyl acetate extracts were excluded from the antimicrobial analysis because systematic optical interference arising from extract pigmentation prevented reliable assessment of microbial growth inhibition.

A clear solvent-dependent pattern of antimicrobial activity was observed for both *S. aureus* and *C. albicans*, with the 70% ethanol extracts showing greater growth inhibition than the *n*-hexane extracts. Against *S. aureus*, the 70% ethanol extracts produced complete growth inhibition at 25 mg/mL for the Pharmacy and Botanical Garden extracts (median 100.0%, Q1–Q3 100.0–100.0 for both) and near-complete growth inhibition for the Wild extract (96.5%, 90.1–97.5). The Botanical Garden 70% ethanol extract retained complete inhibition down to 12.5 mg/mL, yielding the lowest inhibitory concentration values among the *S. aureus* results (MIC_50_ = MIC_90_ = 12.5 mg/mL), whereas the Pharmacy 70% ethanol extract reached MIC_50_ at 12.5 mg/mL and MIC_90_ at 25 mg/mL, and the Wild 70% ethanol extract reached both thresholds only at 25 mg/mL. In contrast, the *n*-hexane extracts achieved only partial inhibition at 25 mg/mL, with medians of 35.5% (Botanical Garden), 46.6% (Pharmacy), and 63.4% (Wild); the MIC_50_ threshold was reached only by the Wild extract (25 mg/mL), and no *n*-hexane extract fulfilled the MIC_90_ criterion within the tested concentration range. The solvent effect was highly significant (Mann–Whitney *U* = 0.0, *p* < 0.001), indicating complete separation between the two solvent groups.

A similar, and in part more pronounced, pattern was observed against *C. albicans*. The 70% ethanol extracts produced complete growth inhibition at 25 mg/mL for all three raw material sources (median 100.0%, Q1–Q3 100.0–100.0). The Botanical Garden 70% ethanol extract retained complete inhibition down to 6.25 mg/mL and ≥50% inhibition down to 3.125 mg/mL (MIC_50_ = 3.125 mg/mL, MIC_90_ = 6.25 mg/mL); the Pharmacy 70% ethanol extract reached both MIC thresholds at 12.5 mg/mL (MIC_50_ = MIC_90_ = 12.5 mg/mL), and the Wild 70% ethanol extract reached MIC_50_ at 3.125 mg/mL and MIC_90_ at 25 mg/mL. In contrast, the *n*-hexane extracts produced only weak to moderate effects at 25 mg/mL (medians 18.8–46.4%) and did not fulfill the monotonic MIC_50_ criterion at any tested concentration. The solvent effect was again highly significant (*U* = 0.0, *p* < 0.001).

None of the tested extracts exhibited notable activity against *K. pneumoniae*. Median growth inhibition at the highest tested concentration (25 mg/mL) ranged from 4.0% (Botanical Garden *n*-hexane extract) to 18.3% (Botanical Garden 70% ethanol extract), and inhibition remained below 20% across the entire tested concentration range (0.195–25 mg/mL) for all six extract–origin combinations (Figure 5 and Table S1). Consequently, neither MIC_50_ nor MIC_90_ could be determined for any extract (MIC > 25 mg/mL), and no statistically significant solvent effect was observed (Mann–Whitney *U* = 14.0, *p* = 0.065).

The origin of the raw material did not significantly influence antimicrobial activity at 25 mg/mL for any of the three test microorganisms (Kruskal–Wallis test: *S. aureus H* = 0.10, *p* = 0.954; *K. pneumoniae H* = 0.08, *p* = 0.960; *C. albicans H* = 1.08, *p* = 0.582). These findings indicate that, under the conditions of this study and among the two extraction solvents included in the antimicrobial analysis, the extraction solvent had a stronger influence on antimicrobial activity than raw material origin.

### 2.6. Cytotoxic Activity of Inula helenium Root Extracts

**Figure 6:**
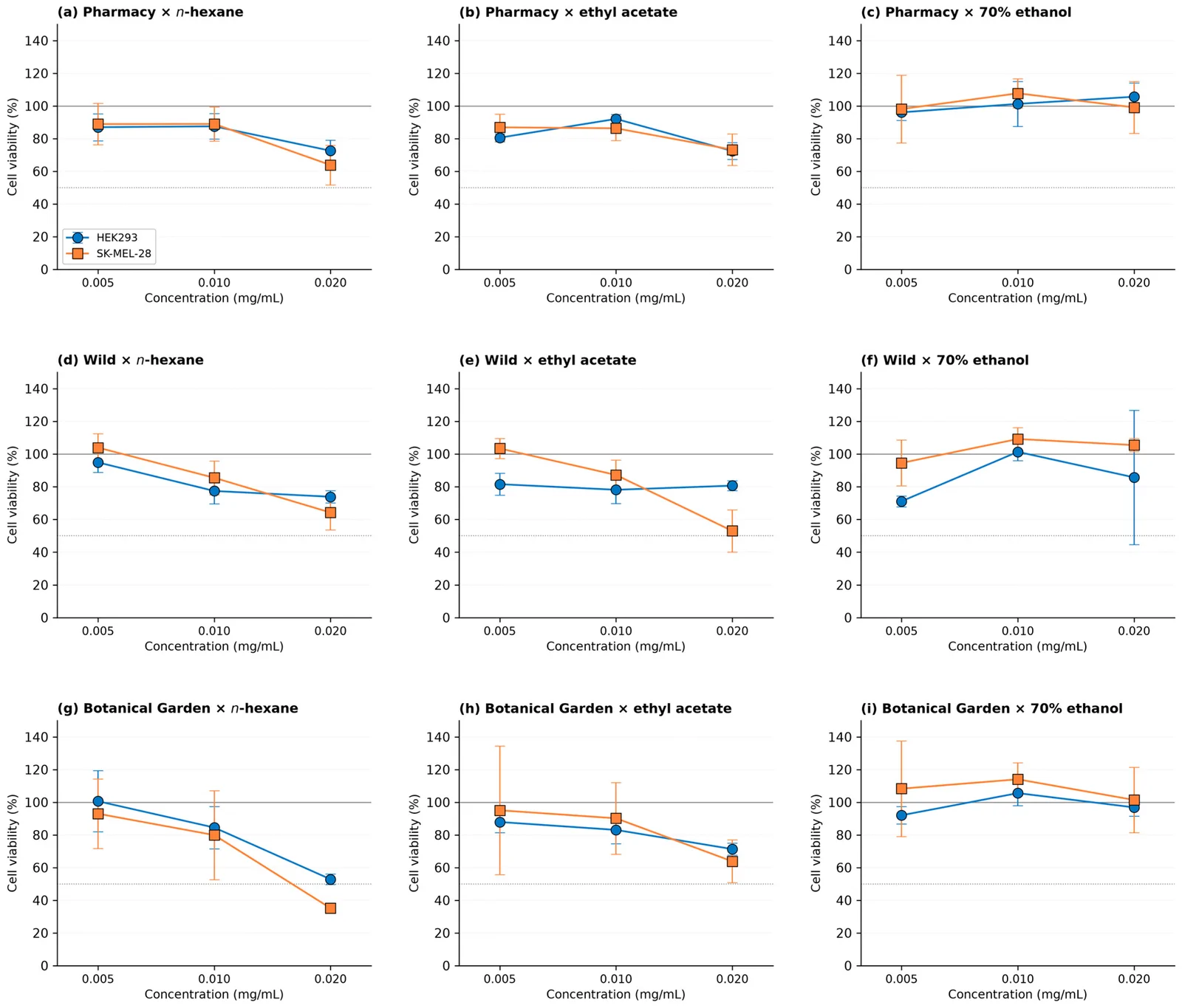
Dose-dependent effects of *Inula helenium* L. root extracts on HEK293 (blue) and SK-MEL-28 (orange) cell viability, assessed by the CCK-8 assay. Values are expressed as mean ± SD of *n* = 3 biological replicates, each measured in technical triplicate. Cell viability was normalized to the DMEM negative control (100%). The dotted line at 50% indicates the threshold for IC_50_ determination. Panels: (**a**–**c**) Pharmacy, (**d**–**f**) Wild, (**g**–**i**) Botanical Garden extracts in *n*-hexane, ethyl acetate, and 70% ethanol, respectively.

The cytotoxic activity of *I. helenium* root extracts was evaluated using the CCK-8 assay in the SK-MEL-28 human melanoma cell line and the HEK293 non-cancerous human embryonic kidney cell line at three concentrations (0.005, 0.010, and 0.020 mg/mL). The positive control (cisplatin, 20 μM) produced a clear reduction in cell viability in both SK-MEL-28 and HEK293 cell lines compared with the vehicle control. The reduction was greater in SK-MEL-28 cells than in HEK293 cells, confirming adequate responsiveness of the assay. Dose-dependent changes in cell viability are presented in Figure 6, whereas the corresponding quantitative data are summarised in Table 4.

**Figure 7:**
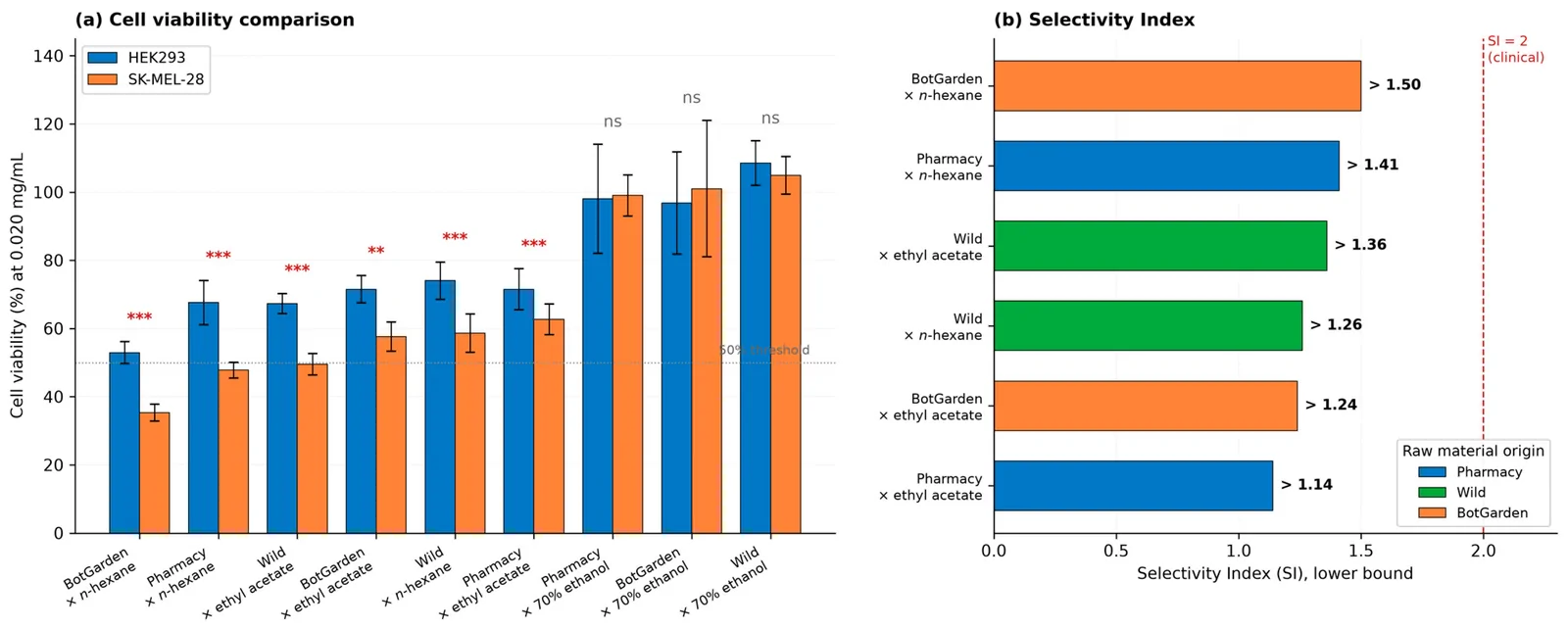
Differential cytotoxicity of *Inula helenium* root extracts in SK-MEL-28 and HEK293 cells at the highest tested concentration (0.020 mg/mL). (**a**) Cell viability comparison between HEK293 (blue) and SK-MEL-28 (orange) cells, ordered by ascending SK-MEL-28 viability. Asterisks indicate significant differences (Mann–Whitney *U* test): *** *p* < 0.001, ** *p* < 0.01, ns = not significant. (**b**) Selectivity Index (SI = HEK293 IC_50_/SK-MEL-28 IC_50_) lower-bound estimates, colour-coded by raw material origin. As HEK293 IC_50_ exceeded 0.020 mg/mL for all extracts, SI values represent lower-bounds. The Botanical Garden *n*-hexane extract demonstrated the strongest preferential cytotoxicity toward SK-MEL-28 cells (SI > 1.50, *p* = 0.0003), followed by the Pharmacy *n*-hexane extract (SI > 1.41) and the Wild ethyl acetate extract (SI > 1.36).

Differential cytotoxicity between HEK293 and SK-MEL-28 cells was additionally summarized using selectivity index (SI = HEK293 IC_50_/SK-MEL-28 IC_50_); the selectivity profile at the highest tested concentration (0.020 mg/mL) and the corresponding selectivity indices are summarised in Figure 7 and Table 5.

*Effect of extraction solvent on cytotoxic activity*. Among the three extraction solvents tested, both *n*-hexane and ethyl acetate extracts produced concentration-dependent cytotoxicity against the two cell lines, whereas 70% ethanol extracts showed no measurable cytotoxicity across all raw material sources (Figure 6 and Table 4). At the highest tested concentration (0.020 mg/mL), 70% ethanol extracts maintained HEK293 viability at 96.8–108.5% and SK-MEL-28 viability at 99.1–104.9%, with no significant differences from the DMEM control. In contrast, *n*-hexane and ethyl acetate extracts produced comparable concentration-dependent reductions in cell viability, reaching SK-MEL-28 viability values of 35.3–58.6% (*n*-hexane) and 49.5–62.7% (ethyl acetate) at 0.020 mg/mL (Table 4).

*Preferential cytotoxicity*. Six of the nine extracts tested displayed statistically significant preferential cytotoxicity against SK-MEL-28 melanoma cells compared with the non-cancerous HEK293 cells at 0.020 mg/mL (Mann–Whitney *U* test, *p* ≤ 0.0012; Figure 7 and Table 5). The Botanical Garden *n*-hexane extract demonstrated the most pronounced melanoma-cell cytotoxicity within the tested concentration range, reducing SK-MEL-28 viability to 35.3 ± 2.5% while HEK293 viability remained at 52.9 ± 3.2% (SI > 1.50; *p* = 0.0003). The Pharmacy *n*-hexane extract showed comparable selectivity (SK-MEL-28: 47.8 ± 2.3%, HEK293: 67.6 ± 6.5%; SI > 1.41; *p* = 0.0004), and the Wild ethyl acetate extract similarly showed preferential cytotoxicity toward SK-MEL-28 melanoma cells (SK-MEL-28: 49.5 ± 3.1%, HEK293: 67.3 ± 2.9%; SI > 1.36; *p* = 0.0007). Because HEK293 viability did not reach 50% in any group within the tested concentration range, classical IC_50_-based SI values could not be determined; therefore, the reported SI values should be interpreted as lower-bound estimates only. *Effect of raw material origin*. Within each solvent class, the raw material source influenced the magnitude, but not the qualitative pattern, of the cytotoxic response. For *n*-hexane extracts, cytotoxicity against SK-MEL-28 cells at 0.020 mg/mL increased in the order Wild (58.6%) < Pharmacy (47.8%) < Botanical Garden (35.3%). Among the ethyl acetate extracts, the Wild source produced the most pronounced preferential cytotoxicity toward SK-MEL-28 cells at 0.020 mg/mL.

### 2.7. Integrated Multivariate Analysis

**Figure 8:**
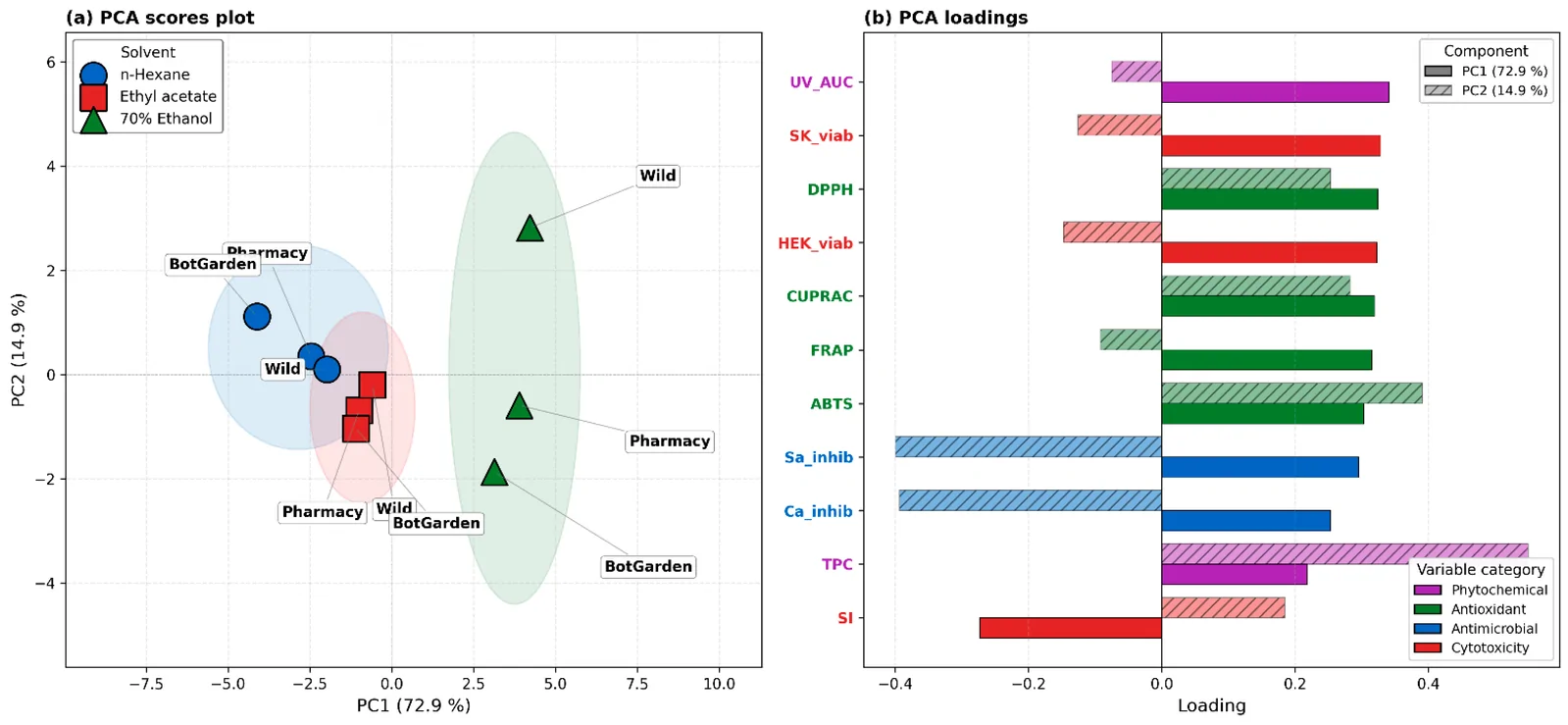
Principal component analysis (PCA) of integrated *Inula helenium* L. root extract bioactivity data (9 samples × 11 variables). (**a**) PCA scores plot showing the distribution of samples in PC1–PC2 space; ellipses indicate clustering by extraction solvent (*n*-hexane, ethyl acetate, 70% ethanol). Sample labels indicate the raw material origin (Pharmacy, Wild, Botanical Garden) connected to data points by leader lines. (**b**) PCA loadings plot showing the contribution of each variable to PC1 and PC2 (solid bars and hatched bars, respectively); variables are color-coded by functional category (Phytochemical, Antioxidant, Antimicrobial, Cytotoxicity). PC1 and PC2 jointly explain 87.9% of total variance. Sa_inhib (*S. aureus* inhibition), Ca_inhib (*C. albicans* inhibition); Cytotoxicity—HEK_viab, SK_viab (HEK293/SK-MEL-28 viability), SI (selectivity index).

**Figure 9:**
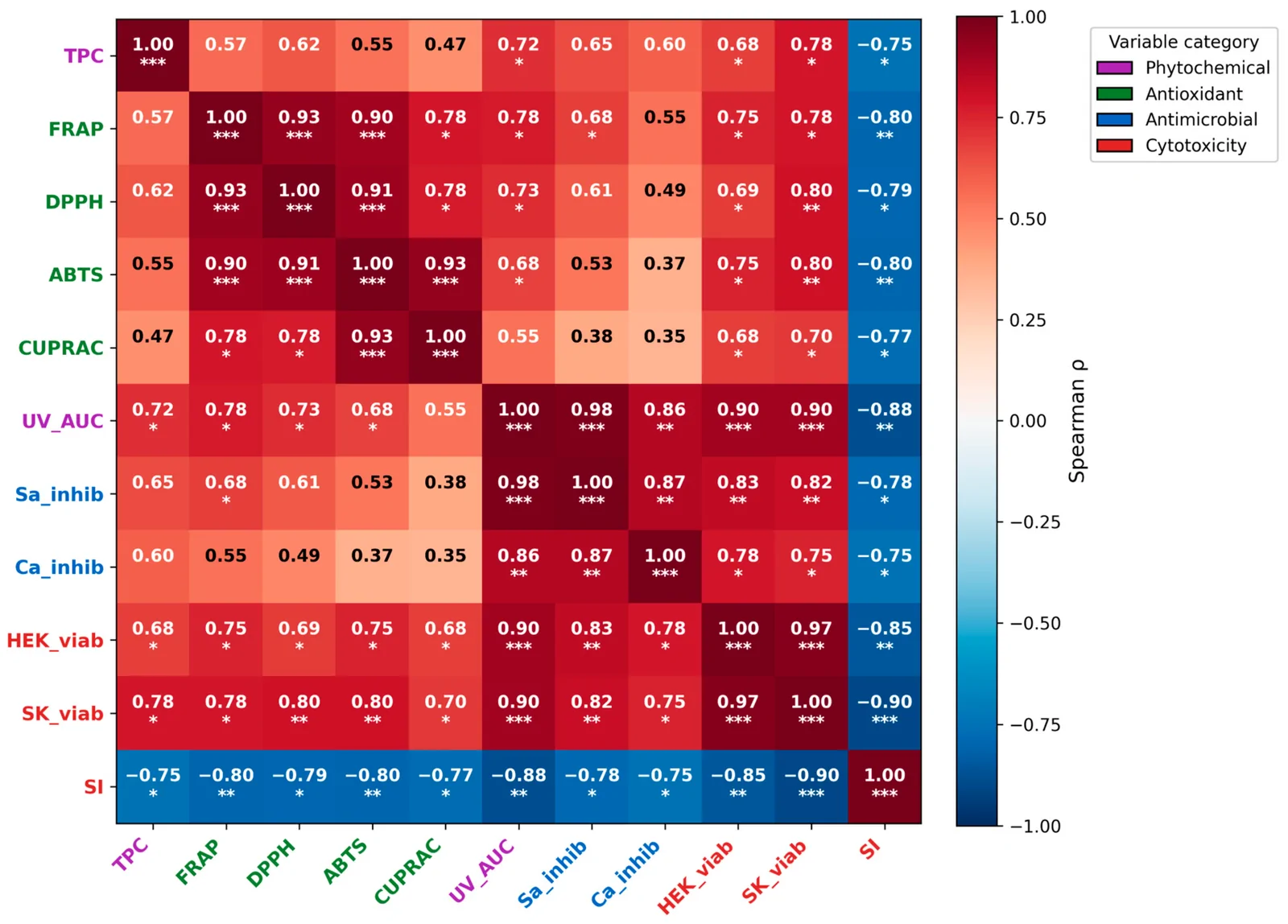
Spearman rank correlation matrix among 11 bioactivity variables (*n* = 9 samples). Cell values represent Spearman *ρ* coefficients with significance indicators (* *p* < 0.05, ** *p* < 0.01, *** *p* < 0.001). Axis labels are color-coded by functional category: Phytochemical (purple)—TPC, UV_AUC; Antioxidant (green)—FRAP, DPPH, ABTS, CUPRAC; Antimicrobial (blue)—Sa_inhib (*S. aureus* inhibition), Ca_inhib (*C. albicans* inhibition); Cytotoxicity (red)—HEK_viab, SK_viab (HEK293/SK-MEL-28 viability), SI (selectivity index).

**Figure 10:**
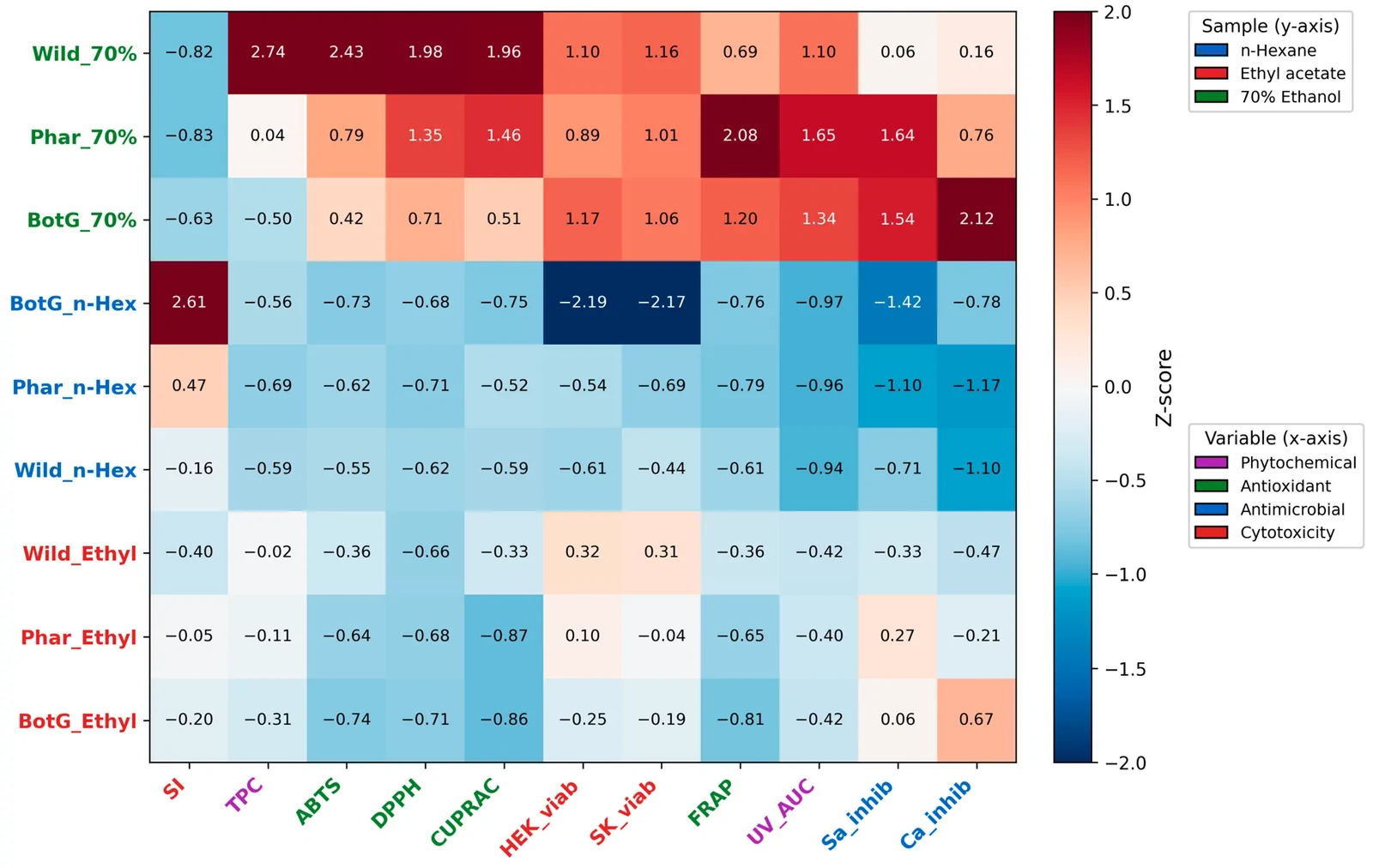
Hierarchical clustering heatmap of *z*-scored bioactivity variables. Rows (samples) and columns (variables) were independently reordered using Ward linkage on Euclidean distances. Cell values indicate *z*-scores (deviation from the across-sample mean). Sample labels (*y*-axis) are color-coded by extraction solvent; variable labels (*x*-axis) by functional category.

An integrated multivariate analysis was performed on the median values of nine origin–solvent groups across eleven bioactivity variables. The dataset included two phytochemical descriptors (TPC and UV_AUC), four antioxidant assays (FRAP, DPPH, ABTS and CUPRAC), antimicrobial activity against *S. aureus* and *C. albicans* at 25 mg/mL (Sa_inhib and Ca_inhib), cell viability of HEK293 (HEK_viab) and SK-MEL-28 (SK_viab) cells, and the selectivity index (SI). *K. pneumoniae* inhibition data were excluded from the integrated dataset because growth inhibition remained below 20% across all evaluated *n*-hexane and 70% ethanol extracts, and neither the MIC_50_ nor the MIC_90_ threshold was reached (Section 2.5). Relationships among variables and extracts were explored using three complementary multivariate approaches: principal component analysis (PCA), Spearman rank correlation, and hierarchical clustering with heatmap visualization (Figure 8, Figure 9 and Figure 10).

Principal component analysis condensed the 11-variable dataset into two principal components that together explained 87.9% of the total variance (PC1 = 75.5%, PC2 = 12.4%; Figure 8a). All phytochemical (TPC, UV_AUC), antioxidant (FRAP, DPPH, ABTS and CUPRAC), antimicrobial (Sa_inhib and Ca_inhib) and cell-viability variables (HEK_viab and SK_viab) loaded positively on PC1, whereas the selectivity index (SI) was the only variable with a negative loading (Figure 8b). PC2 was driven primarily by the Wild 70% ethanol extract, which was clearly separated from the remaining ethanolic extracts. The PCA score plot revealed three distinct, non-overlapping clusters corresponding to the extraction solvent (*n*-hexane, ethyl acetate and 70% ethanol), whereas the raw material source produced only minor within-cluster variation.

The Spearman correlation matrix (Figure 9) revealed strong positive correlations among the four antioxidant assays (*ρ* = 0.78–0.93, *p* ≤ 0.014). The UV–Vis area under the curve (UV_AUC) showed moderate to strong positive correlations with the antioxidant assays (*ρ* = 0.55–0.78), three of which reached statistical significance. Total phenolic content (TPC) was moderately correlated with the remaining variables (*ρ* = 0.47–0.78), with significant positive associations observed for UV_AUC and both cell viability measures. Both antimicrobial activity descriptors (*S. aureus* and *C. albicans* growth inhibition) were positively associated with the phytochemical, antioxidant, and cell viability variables, with generally stronger correlations observed for *S. aureus* inhibition than for *C. albicans* inhibition. HEK293 and SK-MEL-28 cell viability were strongly inter-correlated (*ρ* = 0.97, *p* < 0.001). In contrast, the selectivity index (SI) showed strong negative correlations with all remaining variables (*ρ* = −0.75 to −0.90, all *p* < 0.05).

The hierarchical clustering heatmap (Figure 10) reproduced the solvent-dependent grouping observed in the PCA score plot. The three 70% ethanol extracts formed a distinct cluster characterized by high *z*-scores for most phytochemical, antioxidant, antimicrobial and cell-viability variables. In contrast, the *n*-hexane extracts clustered separately and were characterized by the highest SI values, together with generally low values for the remaining variables. The ethyl acetate extracts occupied an intermediate position between these two clusters. Variable clustering grouped the phytochemical, antioxidant, antimicrobial and cell-viability descriptors together, whereas SI formed a distinct, separate branch.

## 3. Discussion

### 3.1. Solvent Polarity as the Dominant Driver of Bioactivity

The multivariate integration of eleven bioactivity variables across the three raw material origins and three extraction solvents revealed a single overarching pattern: solvent polarity—and not the geographic or commercial source of the plant material—was the predominant determinant of the phytochemical and biological profile of *Inula helenium* root extracts under the experimental conditions of this study. Principal component analysis condensed 87.9% of the total variance into PC1 + PC2, with samples segregating exclusively by extraction solvent while raw material origin contributed only minor intra-cluster displacement (Section 2.7, Figure 8). Mechanistically, this segregation reflects the well-established principle that extraction efficiency is governed by the match between solvent polarity and the polarity of the target constituents, so that *n*-hexane and ethyl acetate preferentially recover the lipophilic sesquiterpene-lactone fraction, whereas 70% ethanol selectively extracts the hydrophilic polyphenolic constituents—two chemical classes that drive fundamentally different biological activities [24]. This pattern closely mirrored the one observed in the UV–Vis spectral fingerprint (Section 2.4, Figure 4), suggesting that differences in the overall extract composition reflected by UV–Vis spectra are associated with the observed variation in antioxidant, antimicrobial, and cytotoxic activities. The dominant influence of solvent polarity observed in the present study is consistent with methodological reviews emphasizing that solvent selection is one of the most influential experimental parameters in phytochemical bioactivity studies [25,26]. A comparable solvent-dependent segregation of bioactivity has been directly reported for *I. helenium* root extracts, where a systematic comparison of 95% ethanol and water extracts across four antioxidant assays revealed markedly higher total phenolic content and antioxidant capacity in the more polar (water) fraction, with water extracts showing approximately two-fold higher TPC and up to three-fold higher FRAP values than the corresponding 95% ethanol extracts obtained from the same raw material [13]. A similar polarity-dependent recovery of polyphenolic constituents has also been documented for the related species *I. viscosa* [27], consistent with the interpretation that solvent polarity is an important determinant of the phytochemical profile within the genus, although further studies across additional Inula species would be needed to establish the generality of this trend. Directly for *I. helenium* roots, an ultrasound-assisted extraction study comparing water, 96.5% ethanol, 50% ethanol and methanol as solvents reported the highest total polyphenol content in 96.5% ethanol extracts (20.05 mg/g DM), while 50% ethanol at a 1:10 plant-to-solvent ratio yielded the strongest overall antioxidant capacity in the CUPRAC assay (2.141 mg/g); the same study confirmed that alantolactone and isoalantolactone together constitute the dominant sesquiterpene-lactone fraction of the raw material (cumulatively ~86% of the GC-MS-identified constituents), alongside a substantial inulin content [28]. These solvent-dependent yield profiles are directly consistent with the compositional differentiation observed in the present study. Taken together, these findings support the use of ultrasound-assisted extraction with solvents of increasing polarity as a rational and reproducible strategy for enriching chemically distinct fractions of bioactive constituents from *I. helenium* roots [29].

### 3.2. Phenolic vs. Non-Phenolic Contributions to Antioxidant Activity

The four antioxidant assays employed in the present study—DPPH, ABTS, FRAP, and CUPRAC—cover different mechanistic aspects of redox activity, ranging from hydrogen-atom and electron-transfer reactions (DPPH) to decolorisation-based electron-transfer (ABTS) and mixed-mode electron-transfer at low or neutral pH (FRAP and CUPRAC) [2,30,31,32]. Despite these mechanistic differences, the four assays produced strongly and mutually correlated results (*ρ* = 0.78–0.93; Section 2.3, Figure 2), indicating that they likely reflect a largely overlapping pool of redox-active constituents. Together, these findings indicate that antioxidant activity in *I. helenium* root extracts cannot be adequately described by total phenolic content alone but rather reflects the combined contribution of multiple classes of redox-active constituents extracted according to solvent polarity. This observation further supports the value of a multi-assay antioxidant approach for the characterization of complex plant extracts, as recommended in recent methodological reviews emphasizing the limitations of relying on a single in vitro antioxidant assay [22,33]. Among the 70% ethanolic extracts, the Wild-collected roots consistently ranked highest in TPC, DPPH, ABTS, and CUPRAC. This observation is consistent with previous reports describing high phenolic content and antioxidant activity of hydroalcoholic *I. helenium* root extracts [8,13], although direct comparisons among wild, cultivated, and commercial raw materials remain scarce. A comparable pattern of high phenolic content coupled with strong antioxidant activity has been reported for hydroalcoholic and ethyl acetate extracts of the related species *Inula viscosa*, where the total phenolic content strongly paralleled DPPH, ABTS, and FRAP responses across solvents of increasing polarity [34], supporting the interpretation that polyphenolic constituents of Inula roots and aerial parts are the primary drivers of radical-scavenging activity in polar extracts.

The FRAP assay, however, produced the inverse ranking (Pharmacy > Botanical Garden > Wild), which is the only qualitative discrepancy across the four methods. This divergence between FRAP and the radical-scavenging assays is a known limitation of single-assay antioxidant characterisation and reinforces the value of a multi-assay approach [2,31]. Consistent with this interpretation, recent comparative work using a library of 375 plant extracts has confirmed that FRAP captures only a specific type of antioxidant activity (reducing power) and can be influenced by matrix components that do not respond in radical-scavenging assays, whereas the Folin–Ciocalteu-based TPC assay is itself susceptible to interference from non-phenolic constituents such as reducing sugars, amino acids and proteins, which can result in TPC overestimation [35]. A similar divergence between reducing-power assays has been directly observed for *I. helenium* root extracts, where CUPRAC values were reported to be approximately ten-fold higher than FRAP values in the same hydroalcoholic sample, further illustrating that these two reducing-power assays capture partly non-overlapping fractions of the antioxidant pool [28].

An additional non-trivial observation is the moderate and non-significant correlation between TPC and the four antioxidant assays (*ρ* = 0.47–0.62; all *p* > 0.05). Two complementary factors can explain this partial dissociation. First, the Folin–Ciocalteu assay is not equally responsive to all phenolic structures and is influenced by their reducing capacity. Simple monohydroxy phenols, ortho-dihydroxy catechols, and methylated derivatives differ markedly in both Folin reactivity and intrinsic radical-scavenging or reducing capacity [36]. Second, *I. helenium* roots are rich in non-phenolic bioactive constituents, most notably sesquiterpene lactones such as alantolactone and isoalantolactone [8,9,10,37], which may also contribute to the overall redox profile of the extracts and, in particular, to reducing capacity measured by FRAP and CUPRAC, although this hypothesis requires confirmation by targeted phytochemical analysis.

The absorbance maxima observed in the UV–Vis fingerprint (211 nm for *n*-hexane, 234–252 nm for ethyl acetate, and 203–327 nm for 70% ethanol extracts; Section 2.4) are consistent with compositional differences between the extracts. Previous chromatographic studies have identified chlorogenic acid and the sesquiterpene lactones alantolactone and isoalantolactone among the constituents of *I. helenium* roots [38], providing relevant phytochemical context for the observed spectral differences. However, UV–Vis spectroscopy alone does not permit assignment of individual absorption bands to specific compounds. The clear solvent-dependent separation observed in PCA and HCA, where samples clustered according to their spectral characteristics and overall bioactivity profiles, further supports polarity-dependent differences in extract composition, while identification of the individual compounds responsible for these spectral features would require compound-specific chromatographic analysis, such as HPLC or LC–MS.

### 3.3. Differential Antimicrobial Activity

The 70% ethanol extracts consistently outperformed the *n*-hexane extracts against both *S. aureus* and *C. albicans* (Section 2.5), producing complete or near-complete growth inhibition of *S. aureus* and complete growth inhibition of *C. albicans* at 25 mg/mL. The lowest MIC_90_ values were 12.5 mg/mL for the Botanical Garden 70% ethanol extract against *S. aureus* and 6.25 mg/mL for the Botanical Garden 70% ethanol extract against *C. albicans*. This activity profile agrees with earlier reports on the antibacterial and antifungal activity of *I. helenium* root preparations [21,39] and is consistent with the traditional use of elecampane for the treatment of microbial infections, particularly those caused by Gram-positive bacteria and fungi [4]. Moreover, the antimicrobial profile closely paralleled the antioxidant profile, with ethanolic extracts consistently exhibiting the highest activity in both assay groups, further supporting the central role of solvent polarity in shaping extract bioactivity. However, because these antimicrobial observations were obtained in a single independent experiment without reference to antimicrobial drug controls, they should be regarded as preliminary and require independent confirmation.

An interesting observation of the present study is that the antimicrobial activity was consistently associated with the polar (70% ethanol) extracts rather than with the lipophilic (*n*-hexane) fractions, which contrasts with previous reports attributing the antibacterial activity of *I. helenium* roots primarily to lipophilic sesquiterpene lactones—most notably alantolactone and isoalantolactone—acting via α,β-unsaturated-γ-butyrolactone-mediated Michael addition to bacterial thiol-containing targets and membrane-disruption mechanisms [4,16]. In these earlier studies, bioactivity-guided fractionation of *I. helenium* root extracts systematically enriched the anti-*Staphylococcus aureus* activity into non-polar sesquiterpene-lactone-rich fractions. In the present work, in contrast, growth inhibition of both *S. aureus* and *C. albicans* segregated with the hydroalcoholic extracts and paralleled the polyphenol-driven antioxidant profile, whereas the *n*-hexane extracts—which by UV–Vis fingerprint and solvent polarity are expected to concentrate the sesquiterpene-lactone fraction—showed substantially weaker antimicrobial activity. Several complementary factors may explain for this apparent divergence: differences in the sesquiterpene-lactone yield obtained by ultrasound-assisted extraction at the concentrations tested here compared with the more exhaustive fractionation protocols used in previous work; a possible additive or synergistic contribution of hydrophilic constituents (phenolic acids, flavonoids and other co-extracted compounds) that are absent from purified lactone fractions; and differences in the microbial strains, inoculum densities and assay formats employed. Together, these observations are consistent with a broader, multi-constituent, polarity-dependent framework that complements the established sesquiterpene-lactone-centered model of *I. helenium* antimicrobial activity, suggesting that hydrophilic phenolic constituents co-extracted under hydroalcoholic conditions may also make a significant contribution, at least under the extraction and delivery conditions used in the present study.

In contrast, the evaluated *n*-hexane and 70% ethanol extracts showed only limited activity against *K. pneumoniae*, with median growth inhibition remaining below 20% across the tested concentration range and reaching a maximum of 18.3% at 25 mg/mL. This limited activity may be partly explained by the permeability barrier of the Gram-negative outer membrane [40]. In *K. pneumoniae*, this barrier is reinforced by additional resistance layers, including reduced-permeability porins, RND-family efflux pumps, and a protective polysaccharide capsule in many clinical isolates [41]. Such mechanisms are likely to contribute to the reduced susceptibility of *K. pneumoniae* to extract constituents that showed activity against Gram-positive bacteria and yeasts in the present study. The clinical relevance of this observation is amplified by the fact that *K. pneumoniae* is currently listed among the WHO priority pathogens for the development of new antimicrobial agents [3]. Nonetheless, recent evidence indicates that plant-derived compounds—including phenolic acids, flavonoids and monoterpenes—can contribute to *K. pneumoniae* control when combined with conventional antibiotics or permeability-enhancing strategies, acting through outer-membrane destabilisation, efflux-pump inhibition and biofilm disruption [42]. Whether *I. helenium* extracts could similarly be repositioned as adjuvants rather than as stand-alone antimicrobials against Gram-negative pathogens remains an open question that merits dedicated combination-based follow-up studies.

In the integrated multivariate analysis, *S. aureus* and *C. albicans* growth inhibition showed the strongest positive correlations with UV_AUC (*ρ* = 0.98 and 0.86, respectively). Their correlations with TPC and the four antioxidant assays were weaker, ranging from *ρ* = 0.38 to 0.68 for *S. aureus* inhibition and from *ρ* = 0.35 to 0.60 for *C. albicans* inhibition. However, because the individual antimicrobial constituents were not identified, these results do not allow attribution of the observed activity to specific phenolic compounds. Rather, these findings suggest that compounds co-extracted under hydroalcoholic extraction conditions—or the combined action of multiple extract constituents—are likely to contribute to both the antioxidant and antimicrobial activities of *I. helenium* roots.

### 3.4. Preferential Cytotoxicity Toward Melanoma Cells

One of the most striking findings of the present study is the pronounced dissociation between antioxidant and cytotoxic activity. Although 70% ethanolic extracts contained the highest phenolic content and displayed the strongest antioxidant activity, they showed no measurable cytotoxicity against either HEK293 or SK-MEL-28 cell lines within the tested concentration range (viability 96.8–108.5%). Conversely, the low-polarity *n*-hexane and ethyl acetate extracts, with negligible phenolic content and antioxidant activity, produced pronounced concentration-dependent cytotoxicity—and, importantly, six of the nine tested extracts displayed statistically significant preferential cytotoxicity against the SK-MEL-28 melanoma cells over the non-cancerous HEK293 cells (Mann–Whitney *U*, all *p* ≤ 0.0012; Section 2.6). Based on lower-bound selectivity index estimates, the Botanical Garden *n*-hexane extract showed the strongest preferential activity toward SK-MEL-28 cells (SI > 1.50; *p* = 0.0003), followed by the Pharmacy *n*-hexane extract (SI > 1.41) and the Wild ethyl acetate extract (SI > 1.36). This mechanistic separation between antioxidant and cytotoxic bioactivities indicates that the hydrophilic phenolic constituents preferentially extracted with 70% ethanol are not the principal cytotoxic agents in *I. helenium* roots. Instead, cytotoxic activity may be associated with lipophilic constituents preferentially extracted with *n*-hexane and ethyl acetate—most notably sesquiterpene lactones such as alantolactone and isoalantolactone, which are among the principal lipophilic constituents of *I. helenium* roots [9,10,11,20]. The molecular basis of the cytotoxic activity of isolated *I. helenium* sesquiterpene lactones has been extensively investigated. Alantolactone and isoalantolactone act primarily through α,β-unsaturated lactone-mediated Michael addition to thiol-containing intracellular targets, leading to the accumulation of reactive oxygen species (ROS), depletion of reduced glutathione, mitochondrial dysfunction, and activation of intrinsic apoptotic pathways [43,44]. Previous studies have demonstrated potent antiproliferative activity of isolated alantolactone and isoalantolactone against a range of cancer cell lines, including gastric (MK-1), cervical (HeLa), murine melanoma (B16F10), ovarian (A2780 and SK-OV-3), and pancreatic (PANC-1) cancer cells [11,20].

Beyond these compound-level observations, evidence that this activity is retained in whole *I. helenium* extracts at the human melanoma level has begun to emerge only recently. A very recent study reported that *I. helenium* extract released from a mineral–botanical nanofibrous scaffold produced selective cytotoxicity against A375 human melanoma cells while sparing healthy human dermal fibroblasts, with the anti-tumoral effect attributed to eudesmane-type sesquiterpene lactones (alantolactone and isoalantolactone) triggering ROS accumulation and simultaneous inhibition of the Wnt/β-catenin and STAT3 signalling cascades [45]. The differential response was explained by the substantially higher baseline enzymatic antioxidant capacity of non-cancerous cells, which allowed them to neutralise the phytochemical-induced oxidative stress that proved lethal for melanoma cells. This tumor-versus-normal-cell dichotomy closely parallels the SK-MEL-28-versus-HEK293 selectivity observed here for the low-polarity *I. helenium* extracts and provides a plausible mechanistic framework for the preferential cytotoxicity documented in the present study. Nonetheless, because the chemical composition of the *n*-hexane and ethyl acetate extracts was not established in the present study, the specific sesquiterpene lactones underlying the observed SK-MEL-28-preferential activity remain to be formally identified by targeted phytochemical analysis. Even so, the clear separation between antioxidant-rich polar extracts and cytotoxic low-polarity extracts provides a strong rationale for future bioassay-guided fractionation, identification of the active constituents, and mechanistic investigations.

### 3.5. Practical Implications for Extraction Strategy and Standardization

Taken together, the present results have direct practical implications for the standardization of *I. helenium* root preparations. Within the experimental conditions of this study, the choice of extraction solvent appeared to exert a substantially greater influence on bioactivity than the geographic or commercial source of the raw material, allowing extraction conditions to be tailored toward a desired biological profile. Specifically, 70% ethanol maximized phenolic content, antioxidant capacity, and antimicrobial activity against Gram-positive bacteria and yeast, whereas *n*-hexane preferentially recovered a lipophilic fraction with preferential in vitro cytotoxicity against melanoma cells.

The similarity of the ethanolic bioactivity profile across the three origins suggests that commercially available, cultivated, and wild-collected roots may all represent suitable raw materials for hydroalcoholic preparations, although confirmation across additional populations, harvest seasons, and cultivation conditions would strengthen this conclusion. Conversely, among the three evaluated sources, cultivated Botanical Garden roots produced the strongest differential cytotoxic response between SK-MEL-28 and HEK293 (viability-based SI > 1.50, lower-bound estimate). This finding suggests that cultivated material may represent a particularly promising source of lipophilic bioactive constituents under the conditions examined in the present study, although the effect of raw material origin did not reach statistical significance (Kruskal–Wallis, ns), and this observation should therefore be interpreted as a trend requiring confirmation in larger sample sets. This observation is presumably consistent with environmental and developmental factors known to modulate sesquiterpene lactone biosynthesis in Asteraceae [7,46,47].

These findings support the traditional use of *I. helenium* root as an antifungal and Gram-positive antibacterial remedy [4] while also revealing a previously underexplored preferential cytotoxic effect of low-polarity root extracts toward SK-MEL-28 cells compared with HEK293 cells. Together, these results provide a rationale for future bioassay-guided isolation and characterization of the lipophilic constituents responsible for this activity, as well as for the development of standardized extraction strategies tailored to specific therapeutic applications.

### 3.6. Study Limitations

Several methodological limitations should be considered when interpreting the present results. First, the sample size was small (*n* = 3 independent biological replicates per origin–solvent combination), and although the observed effects of extraction solvent were highly significant (Kruskal–Wallis *p* ≤ 0.004; *ε*^2^ = 0.58–0.66), pairwise comparisons among the nine origin–solvent groups did not survive Bonferroni correction in any assay. The consistent ordering of extracts across four independent antioxidant assays, however, supports the biological reality of the observed origin-dependent trends. Nevertheless, these findings should be interpreted with appropriate caution until confirmed in larger datasets. Second, the ethyl acetate extracts were excluded from the antimicrobial dataset because of systematic optical interference caused by extract pigmentation, which produced biologically implausible negative inhibition values across all sample origins and could not be resolved by extract-blank correction. Although this prevented evaluation of the intermediate-polarity fraction, exclusion of these data avoided overinterpretation of technically unreliable measurements. In addition, no reference antibacterial or antifungal agent was included as an antimicrobial positive control. The antimicrobial assay was performed as a single independent experiment with three technical replicate wells per condition and was not independently confirmed using a complementary non-optical method. Although concentration-matched DMSO controls were used at each extract concentration, the relatively high DMSO concentration at the highest extract concentration (5%, *v*/*v*) may have influenced baseline microbial growth and, therefore, represents an additional methodological limitation. Accordingly, the antimicrobial findings should be regarded as preliminary and require independent validation using appropriate reference antimicrobial controls and, preferably, a complementary non-optical viability method.

Third, the phytochemical profile of the extracts was characterized at the fingerprint level (UV–Vis spectra, TPC, AUC) rather than by individual compound identification. Targeted quantification of alantolactone, isoalantolactone, and other sesquiterpene lactones by chromatographic methods (HPLC, LC–MS) would be required to link the observed *n*-hexane cytotoxicity to specific molecular constituents [37]. Similarly, the associations observed between antioxidant, antimicrobial, and cytotoxic activities should be regarded as hypothesis-generating until supported by targeted phytochemical characterization and bioassay-guided fractionation. Fourth, the cytotoxic activity was assessed on only two cell lines (HEK293 and SK-MEL-28) and at three concentrations (0.005–0.020 mg/mL); a broader concentration range and a wider panel of cancer and non-cancer cell lines would be required to establish the therapeutic index of the most promising extracts. Furthermore, a limited concentration range restricted accurate IC_50_ determination for several extracts and did not allow complete characterization of the dose–response relationship. In addition, although HEK293 cells provide a commonly used non-cancerous comparator, validation in additional non-transformed human cell models would further strengthen assessment of differential cytotoxicity between cancerous and non-cancerous cells. Finally, the multivariate patterns observed here are based on nine samples, which limits the statistical power of PCA and hierarchical clustering; expanding the sample size across multiple growing seasons, geographic regions, and cultivation conditions would strengthen the generalization of these findings. Despite these limitations, the convergence of multiple independent analytical approaches—including four antioxidant assays, antimicrobial testing, cytotoxicity assessment, UV–Vis fingerprinting, and multivariate analysis—provides consistent evidence supporting solvent polarity as the principal factor shaping the bioactivity profile of *I. helenium* root extracts under the experimental conditions of this study.

## 4. Materials and Methods

### 4.1. Plant Material, Sampling Sites, and Sample Preparation

**Figure 11:**
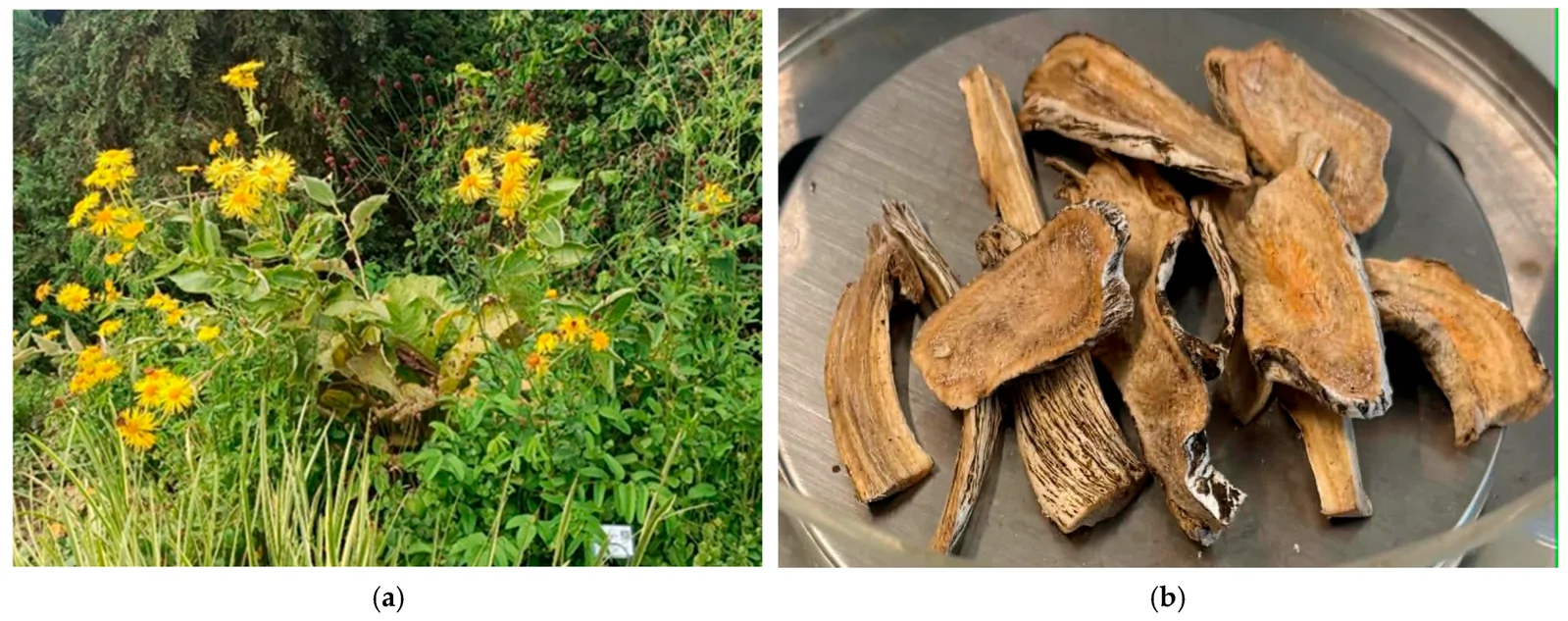
*Inula helenium* L. used as the source of raw material in the present study. (**a**) Flowering plants growing at the Botanical Garden of Vilnius University (Kairėnai, Lithuania) during the vegetative season; (**b**) Sliced and air-dried roots ready for milling and extraction.

Roots of elecampane (*Inula helenium* L., Asteraceae; Figure 11) were obtained from three independent sources to evaluate the influence of origin on extract composition and bioactivity: (i) commercial source (Pharmacy): commercially distributed dried elecampane root tea purchased from a Lithuanian pharmacy [“Švenčionių vaistažolių fabrikas”, Lithuania, batch No. L222307]; three packages of the commercial product were pooled to form a single representative commercial sample; (ii) Wild populations (Wild): roots collected from three natural habitats in the Ignalina district, Lithuania: Mėčionys (55.270107° N, 26.498616° E), Ryžiškė (55.283536° N, 26.510016° E), and Mielagėnai (55.261106° N, 26.438764° E). Samples from all three sites were pooled to form a single representative wild-population sample; (iii) Cultivated source (Botanical Garden): roots collected at the Botanical Garden of Vilnius University, located in Kairėnai, Vilnius district (54.733980° N, 25.405059° E).

Roots from the Wild and Botanical Garden sources were collected during the second half of October 2025 (12 October 2025 for the Wild populations and 7 October 2025 for the Botanical Garden), at the end of the vegetative period, when the accumulation of bioactive compounds in elecampane roots is reported to reach its seasonal maximum [13]. Immediately after collection, freshly harvested roots (Wild and Botanical Garden samples) were washed, sliced thinly, and air-dried on paper sheets at room temperature in a dark, well-ventilated room, away from direct sunlight, until constant weight was reached. The commercial sample was used as supplied. All three samples were then ground with an analytical mill and sieved through a 500 µm mesh sieve. The resulting powders were stored in amber glass bottles at 4 °C until extraction.

### 4.2. Chemicals and Reagents

The following reagents were used in the present study: Folin–Ciocalteu reagent (2 M), gallic acid, Trolox, ABTS, DPPH, potassium persulfate, TPTZ, neocuproine, copper (II) chloride dihydrate, iron (III) chloride, ammonium acetate, sodium acetate trihydrate, sodium carbonate, hydrochloric acid, glacial acetic acid, phosphate-buffered saline tablets, dimethyl sulfoxide (DMSO), trypan blue, *n*-hexane, ethyl acetate, ethanol (≥99.8% and 70%, *v*/*v*), and deionized water. Cisplatin was used as a positive cytotoxic control.

Brain Heart Infusion (BHI) broth and agar were used for the cultivation of the bacterial strains, whereas Sabouraud dextrose agar was used for the cultivation of *C. albicans*. The ATCC-derived reference strains *S. aureus* ATCC 25923, *K. pneumoniae* ATCC BAA-1705, and *C. albicans* ATCC 10231, as well as all culture media, were obtained from Liofilchem S.r.l. (Roseto degli Abruzzi, Italy).

The human melanoma SK-MEL-28 and non-cancerous human embryonic kidney HEK293 cell lines were obtained from Cytion GmbH (Berlin, Germany). For cell culture and cytotoxicity assays, Dulbecco’s Modified Eagle Medium (DMEM; Life Technologies, Carlsbad, CA, USA), fetal bovine serum (FBS; Biological Industries, Kibbutz Beit-Haemek, Israel), penicillin–streptomycin solution (P/S; Lonza, Verviers, Belgium), 0.25% trypsin–0.1% EDTA solution, Dulbecco’s phosphate-buffered saline (DPBS), and the Cell Counting Kit-8 (CCK-8; Dojindo Laboratories, Kumamoto, Japan) were used.

Unless otherwise stated, reagents were of analytical grade and purchased from Sigma-Aldrich (St. Louis, MO, USA), Merck (Darmstadt, Germany), Fluka (Buchs, Switzerland), Riedel-de Haën (Seelze, Germany), VWR Chemicals (Radnor, PA, USA), Biological Industries (Kibbutz Beit-Haemek, Israel), Lonza (Verviers, Belgium), and B. Braun Medical AG (Sempach, Switzerland).

### 4.3. Instrumentation

*Sample processing*. Plant material was processed using an analytical mill (SM-450L, MRC Grinding Machine, Holon, Israel), a 500 µm sieve (Labbox, Barcelona, Spain), an analytical balance (AS R2 PLUS, Radwag, Radom, Poland), and a vortex mixer (Vortex V-1 plus, BioSan, Riga, Latvia).

*Extraction and sample preparation*. Extraction was performed using an ultrasonic bath (WUC-A02H, Witeg, Wertheim, Germany), a refrigerated centrifuge (Rotina 380 R, Hettich, Tuttlingen, Germany), a rotary evaporator equipped with a heating bath (IKA RV 10 digital and IKA HB 10 digital, IKA-Werke, Staufen, Germany), and a thermostatic oven (Ecocell 22, MMM Medcenter Einrichtungen GmbH, Munich, Germany) used together with a vacuum desiccator (VWR Chemicals, Leuven, Belgium). Buffer solutions were adjusted with a pH-meter (ClearLine EZ-7, Dominique Dutscher SAS, Bernolsheim, France). Extracts were filtered through 0.22 µm syringe filters (ClearLine, Dominique Dutscher SAS, Bernolsheim, France).

*Spectrophotometry*. Spectrophotometric assays were performed on a UV–Vis spectrophotometer (UV-1600PC, Thermo Scientific, Singapore City, Singapore) using quartz cuvettes (100-QS, Hellma Analytics, Müllheim, Germany), and on a UV–Vis microplate reader (Multiskan Sky, Thermo Scientific, Vantaa, Finland) with 96-well microplates (BRANDplates, Brand, Wertheim, Germany).

*Microbiology*. Microbiological work was carried out using thermostatic oven (Ecocell 22, MMM Medcenter Einrichtungen GmbH, Munich, Germany), an autoclave (T-Edge10, Tuttnauer, Breda, The Netherlands), and a McFarland densitometer (DEN-1B, BioSan, Riga, Latvia) in a Class II biosafety cabinet (Esco Lifesciences, Singapore).

*Cell culture*. Cell-culture procedures were performed in a CO_2_ incubator (CCL-170B-8-CU, Esco Lifesciences, Singapore). Cell morphology was monitored using an inverted microscope (EVOS XL Core, Invitrogen/Thermo Scientific, Bothell, WA, USA) and a live-cell imaging system (Celloger Mini Plus, Curiosis Inc., Seoul, Republic of Korea). Absorbance measurements for the CCK-8 cytotoxicity assay were performed using a Tecan Infinite 200Pro microplate reader (Tecan Group Ltd., Männedorf, Switzerland).

### 4.4. Extract Preparation

Extracts were prepared by ultrasound-assisted extraction (UAE), based on the protocol of Kschonsek et al. [33,48], with two principal modifications tailored to the lipophilic-rich composition of *I. helenium* roots: (i) the acid/base hydrolysis step preceding extraction was omitted, to preserve the native phytochemical composition of the extracts, including sesquiterpene lactones and non-hydrolyzable phenolic constituents, characteristic of this species [13,21,29]; and (ii) instead of methanol/water mixtures, three solvents of increasing polarity were used in parallel to obtain extracts with distinct phytochemical profiles: *n*-hexane (non-polar), ethyl acetate (medium polarity), and 70% (*v*/*v*) aqueous ethanol (polar) [25,26].

For each of the three plant sources (Pharmacy, Wild, Botanical Garden) and each of the three solvents, 6.00 ± 0.10 g of dried, ground and sieved root powder was weighed into a flask and subjected to three consecutive UAE cycles at ≤40 °C, preserving the solid-to-solvent ratio of 1:25 (*w*/*v*) reported by Kschonsek et al. [48]. The first extraction was performed with 60 mL of solvent for 20 min, followed by a second cycle with 60 mL of fresh solvent for 10 min, and a third cycle with 30 mL of fresh solvent for 10 min. After each cycle, the suspensions were centrifuged at 3000× *g* for 5 min, and the supernatants were collected. The three supernatants from a given sample–solvent combination were combined for further processing. Each biological replicate represented an independently extracted sample prepared from a separate portion of homogenized plant material. The complete extraction procedure was performed in triplicate (*n* = 3 independent biological replicates).

The combined extracts were filtered through 0.22 µm syringe filters. The bulk of the solvent was then removed under reduced pressure on a rotary evaporator at 40 °C with a rotation speed of up to 150 rpm. To eliminate residual traces of solvent, the concentrated residues were subsequently transferred to a vacuum desiccator placed in a thermostatic oven (37 °C) and dried until constant mass was reached. The resulting solvent-free dry extracts were stored in amber glass vials at 4 °C protected from light.

For bioactivity assays, the dry extracts were reconstituted at a concentration of 10 mg/mL in 70% ethanol (for total phenolic content, antioxidant assays, and UV–Vis fingerprinting of polar and medium-polarity extracts) or in DMSO (for *n*-hexane extracts, antimicrobial assays, and cytotoxicity assays). Dissolution was facilitated by vortexing and short sonication. The stock solutions were further diluted as required by each individual assay (see Section 4.5, Section 4.6, Section 4.7, Section 4.8 and Section 4.9). The final concentration of DMSO or ethanol in each assay did not exceed the corresponding solvent concentration used in the respective negative controls.

### 4.5. UV–Vis Spectral Fingerprinting

The phytochemical profile of each extract was characterized by recording its full UV–Vis absorbance spectrum. Dry extracts were dissolved in their respective extraction solvent at concentrations adjusted to keep the absorbance within the linear range of the instrument: 0.02 mg/mL for *n*-hexane extracts and 0.20 mg/mL for ethyl acetate and 70% ethanol extracts. These concentrations were selected following preliminary optimization to ensure adequate spectral resolution while avoiding detector saturation. The solutions were transferred to a 1 cm quartz cuvette, and absorbance was scanned from 200 to 800 nm with a 1 nm step on a UV-1600PC spectrophotometer. The respective neat solvent was used as a blank for baseline correction.

For each extract, the wavelengths of local absorbance maxima (*λ*_max_) were identified using a peak-prominence threshold of ≥0.03 AU. The area under the curve (AUC) was integrated using the trapezoidal rule over 200–350 nm for *n*-hexane extracts (no significant absorbance was observed above 350 nm) and over 200–450 nm for ethyl acetate and 70% ethanol extracts. The AUC provided a quantitative descriptor of the overall absorbance intensity across the selected wavelength range and was subsequently used for comparative analysis of the spectral profiles.

### 4.6. Total Phenolic Content (TPC) by Folin–Ciocalteu Assay

Total phenolic content (TPC) of the *I. helenium* root extracts was determined using the Folin–Ciocalteu assay [48,49] following standardized microplate methodology and reporting recommendations [36]. The working Folin–Ciocalteu reagent was prepared by diluting the 2.0 M stock 1:10 (*v*/*v*) with deionized water. Sodium carbonate (7.5% *w*/*v*) was prepared by dissolving 3.75 g Na_2_CO_3_ in deionized water and adjusting the volume to 50.0 mL. A gallic acid stock solution was prepared by dissolving 18.8 mg of gallic acid in deionized water to a final volume of 50 mL. Working calibration standards were obtained by serial dilution of the stock with deionized water at four concentration levels (17.01, 34.02, 85.06, and 170.10 mg/L), yielding the linear regression equation *y* = 0.009490·*x* − 0.04439 (*R*^2^ = 0.9950). In a 96-well plate, 30.0 µL of calibration standards or extracts at four dilution levels (1:1, 1:2, 1:5, and 1:10) were combined with 150.0 µL of working Folin–Ciocalteu reagent and 120.0 µL of 7.5% Na_2_CO_3_. Each standard and each sample dilution was measured in triplicate. Plates were incubated for 2.0 h at room temperature protected from light, and absorbance was recorded at 740 nm after a 5 min equilibration at 30 °C.

A reagent blank and a corresponding sample blank (prepared without the Folin–Ciocalteu reagent) were analysed in parallel, and absorbance values were corrected accordingly before quantification. Corrected absorbance values were converted to gallic acid concentrations using the calibration curve, and TPC was expressed as milligrams of gallic acid equivalents per gram of dry extract (mg GAE/g DE).

### 4.7. Antioxidant Activity Assays

The antioxidant activity of the extracts was evaluated by four complementary spectrophotometric assays—DPPH, ABTS, FRAP, and CUPRAC—performed in 96-well microplates. These assays were selected because they rely on complementary antioxidant reaction mechanisms, including hydrogen atom transfer and single-electron transfer, thereby providing a broader assessment of antioxidant capacity than any individual assay alone [2,30,33]. For all antioxidant assays, both a reagent blank and a corresponding sample blank were analysed in parallel. The reagent blank was used to correct for the intrinsic absorbance of the assay reagents, while the sample blank was used to correct for the intrinsic absorbance of the extract at the detection wavelength. Sample blanks were prepared by omitting the reaction-initiating or chromogenic component of the respective assay (e.g., DPPH, ABTS^+^, or CuCl_2_, as appropriate), whereas reagent blanks contained the complete assay mixture without extract. The corrected absorbance value (*A*_corr_) used for quantification was calculated as:

(1) $$A_{corr}=A_{sample}-A_{sample\;blank}-A_{reagent\;blank},$$

where *A*_sample_ is the absorbance of the extract–reagent mixture, *A*_sample blank_ is the absorbance of the extract measured in the absence of the reaction-initiating or chromogenic reagent, and *A*_reagent blank_ is the absorbance of the complete assay mixture without extract.

Trolox calibration curves (*R*^2^ ≥ 0.99) were prepared for all assays; the calibration range was 12.5–250 µmol/L for the DPPH, ABTS, and CUPRAC assays, and 12.5–200 µmol/L for the FRAP assay. Results were expressed as micromoles of Trolox equivalents per gram of dry extract (µmol TE/g DE), using the general expression:

(2) $$Antioxidant\;activity\left(\mu \mathrm{mol}\;\mathrm{TE}/g\;\mathrm{DE}\right)=(A_{corr}\times DF)/(slope\times C_{extract}),$$

where *A*_corr_ is the corrected absorbance obtained from Equation (1), DF is the dilution factor of the extract, slope is the slope of the Trolox calibration curve, and *C*_extract_ is the extract concentration expressed as g of dry extract per mL. All measurements were performed in triplicate.

#### 4.7.1. DPPH Radical Scavenging Assay

The DPPH assay was performed according to the method of Brand-Williams et al. [50], with minor modifications. A 0.25 mM DPPH solution was freshly prepared by dissolving 5.0 mg of DPPH in 50 mL of ≥99.8% ethanol. For analysis, 100 µL of extract (appropriately diluted in 70% ethanol to fall within the linear range of the Trolox calibration curve) was mixed with 200 µL of DPPH working solution and incubated in the dark at room temperature for 30 min. Absorbance was measured at 515 nm, and the DPPH radical-scavenging activity was calculated as:

(3) $$DPPH\;scavenging\left(\%\right)=[(A_{control}-A_{corr})/A_{control}]\times 100,$$

where *A*_control_ is the absorbance of the DPPH working solution measured against ethanol, and *A*_corr_ is the corrected sample absorbance calculated according to Equation (1). The scavenging percentages were then converted to Trolox equivalents (µmol TE/g DE) using the Trolox calibration curve and Equation (2).

#### 4.7.2. ABTS Radical Cation Decolorization Assay

The ABTS radical cation decolorization assay was performed according to the method of Re et al. [51], with minor modifications. The ABTS^+^ radical cation was generated by mixing equal volumes of 7 mM ABTS aqueous solution and 2.45 mM potassium persulfate solution, followed by incubation in the dark at room temperature for 16–24 h. Prior to use, the stock radical solution was diluted with 10 mM phosphate-buffered saline (pH 7.4) until the absorbance at 730 nm reached 0.70 ± 0.02 AU. For analysis, 20 µL of extract (appropriately diluted to fall within the linear range of the Trolox calibration curve) was mixed with 200 µL of ABTS^+^ working solution and incubated at 30 °C in the dark for 30 min. Absorbance was measured at 730 nm, and the ABTS^+^ radical scavenging activity was calculated as:

(4) $$ABTS\;scavenging(\%)=[(A_{control}-A_{corr})/A_{control}]\times 100,$$

where *A*_control_ is the absorbance of the ABTS^+^ working solution measured against phosphate-buffered saline, and *A*_corr_ is the corrected sample absorbance calculated according to Equation (1). The scavenging percentages were then converted to Trolox equivalents (µmol TE/g DE) using the Trolox calibration curve and Equation (2).

#### 4.7.3. Ferric Reducing Antioxidant Power (FRAP) Assay

The FRAP assay was performed according to the method of Benzie and Strain [52], with minor modifications. The FRAP working reagent was prepared fresh by mixing 300 mM acetate buffer (pH 3.6), 10 mM TPTZ solution in 40 mM HCl, and 20 mM aqueous FeCl_3_ in a 10:1:1 (*v*/*v*/*v*) ratio. For analysis, 30 µL of extract (appropriately diluted to fall within the linear range of the Trolox calibration curve) was mixed with 200 µL of FRAP working reagent in a 96-well microplate, incubated at 37 °C for 30 min in the dark, and absorbance was measured at 593 nm. Because FRAP is based on the direct reduction of the Fe^3+^–TPTZ complex to Fe^2+^–TPTZ, the corrected sample absorbance (*A*_corr_) calculated according to Equation (1) was converted directly into Trolox equivalents (µmol TE/g DE) using the Trolox calibration curve and Equation (2), without an intermediate percentage-based expression.

#### 4.7.4. Cupric Reducing Antioxidant Capacity (CUPRAC) Assay

The CUPRAC assay was performed according to the method of Apak et al. [53], with minor modifications. The reaction mixture contained 20 µL of extract (appropriately diluted to fall within the linear range of the Trolox calibration curve), 50 µL of 10 mM CuCl_2_·2H_2_O, 50 µL of 7.5 mM neocuproine in ethanol, and 60 µL of 1 M ammonium acetate buffer (pH 7.0), made up to a final volume of 200 µL with deionized water. After 30 min of incubation at room temperature in the dark, absorbance was measured at 450 nm. A reagent blank and a corresponding sample blank (prepared without CuCl_2_) were analysed in parallel, and absorbance values were corrected accordingly before quantification. Analogously to FRAP, the corrected sample absorbance (*A*_corr_) calculated according to Equation (1) was converted directly into Trolox equivalents (µmol TE/g DE) using the Trolox calibration curve and Equation (2), without an intermediate percentage-based expression.

### 4.8. Antimicrobial Activity Assay

The antimicrobial activity of *I. helenium* root extracts was evaluated using a broth microdilution assay in 96-well microplates, based on previously published protocols for plant extracts [21,39]. BHI broth was used as the test medium for both the bacterial strains and *C. albicans*. Inoculum densities and selected incubation parameters were established with reference to standardized broth microdilution methods for bacteria and yeasts [54,55]. Three reference strains were tested: *Staphylococcus aureus* ATCC 25923, representing Gram-positive bacteria; *Klebsiella pneumoniae* ATCC BAA-1705, representing Gram-negative bacteria; and *Candida albicans* ATCC 10231, representing yeasts.

*Culture preparation*. Prior to the antimicrobial activity assay, frozen stock cultures of the reference bacterial strains were streaked on BHI agar and incubated at 35 ± 1 °C for 24 h. Several well-isolated colonies were suspended in sterile 0.9% (*w*/*v*) NaCl solution, and the turbidity was adjusted to a 0.5 McFarland standard, corresponding to approximately 1.5 × 10^8^ CFU/mL. The turbidity of each suspension was verified using a McFarland densitometer. The standardized suspensions were subsequently diluted 1:100 in BHI broth to obtain an estimated working concentration of approximately 1.5 × 10^6^ CFU/mL.

A frozen stock culture of *Candida albicans* ATCC 10231 was streaked on Sabouraud dextrose agar and incubated in ambient air at 35 ± 1 °C for 24 h. Five well-isolated colonies were suspended in sterile distilled water, and the turbidity was adjusted to a 0.5 McFarland standard, corresponding to approximately (1–5) × 10^6^ CFU/mL, using a McFarland densitometer. The standardized suspension was subsequently diluted 1:10 in BHI broth to obtain an estimated working inoculum concentration of (1–5) × 10^5^ CFU/mL.

*Extract preparation*. Dry extracts were first dissolved in dimethyl sulfoxide (DMSO) and then diluted with BHI broth to obtain a working stock solution of 50 mg/mL in 10% (*v*/*v*) DMSO. Twofold serial dilutions of this working stock were subsequently prepared in BHI broth to yield eight final extract concentrations in the assay wells (after 1:1 dilution with the microbial suspension): 25, 12.5, 6.25, 3.125, 1.5625, 0.78125, 0.390625, and 0.1953125 mg/mL, with correspondingly decreasing final DMSO concentrations of 5%, 2.5%, 1.25%, 0.625%, 0.3125%, 0.15625%, 0.078125%, and 0.0390625% (*v*/*v*), respectively. Ethyl acetate extracts were excluded from further antimicrobial analyses due to systematic optical interference arising from extract pigmentation, which produced biologically implausible negative inhibition values across all sample origins and could not be reliably corrected using the corresponding extract-background controls.

*Assay procedure*. The antimicrobial assay was performed in 96-well microplates. Each test well contained 100 μL of the corresponding extract dilution and 100 μL of the appropriate microbial suspension, resulting in a final volume of 200 μL. The estimated final inoculum concentrations were approximately 7.5 × 10^5^ CFU/mL for the bacterial strains and (0.5–2.5) × 10^5^ CFU/mL for *C. albicans*.

The following controls were included: (i) a positive growth control containing BHI broth and the corresponding microbial suspension; (ii) a sterility control containing BHI broth without microorganisms; (iii) concentration-matched DMSO controls containing the corresponding microbial suspension but no extract, prepared at the same DMSO concentrations as the extract dilutions (5%, 2.5%, 1.25%, 0.625%, 0.3125%, 0.15625%, 0.078125%, and 0.0390625% (*v*/*v*)); and (iv) extract background controls without microorganisms. No reference antibacterial or antifungal agent was included as an antimicrobial positive control. Because DMSO can exert concentration-dependent effects on microbial growth, particularly on *C. albicans* [56,57], microbial growth inhibition was subsequently calculated for each well relative to the corresponding concentration-matched DMSO control, thereby accounting for the potential contribution of DMSO to the calculated extract-specific antimicrobial effect. Each extract–microorganism combination was tested in three technical replicate wells within a single independent experiment (*n* = 3 technical replicates per condition).

Plates inoculated with *S. aureus* and *K. pneumoniae* were incubated at 35 ± 1 °C for 18 h, whereas plates inoculated with *C. albicans* were incubated under the same temperature conditions for 24 h. Absorbance was measured at 600 nm at room temperature using a microplate reader.

*Data analysis.* Absorbance values from the test wells were corrected by subtracting the absorbance of the corresponding extract background controls, whereas the absorbance values of the DMSO solvent controls were corrected using the sterility control. Microbial growth inhibition (%) was then calculated for each well relative to the corresponding concentration-matched DMSO control, thereby accounting for the potential growth-modulating effect of DMSO at each extract concentration. Individual per-well inhibition values were subsequently capped at the [0, 100] interval prior to summary and statistical analysis, in accordance with the biological definition of the assay: negative raw values (reflecting apparent absorbance greater than the matched DMSO control, i.e., no measurable inhibition) were assigned a value of 0%, and raw values exceeding 100% (attributable to residual absorbance from pigmented extracts) were assigned a value of 100% and interpreted as complete growth inhibition.

MIC_50_ and MIC_90_ were defined as the lowest tested concentration at which the median growth inhibition reached ≥50% and ≥90%, respectively, and at which all higher tested concentrations also met the same threshold (monotonic dose–response criterion). This constraint prevents the assignment of MIC values based on isolated inhibition peaks at low concentrations, which are commonly attributed to measurement artifacts (residual pigment absorbance, DMSO-related solvent effects, or well-to-well variability) rather than genuine antimicrobial activity, in accordance with the monotonicity assumption underlying standard dose–response analysis [58]. When the threshold was not reached at any tested concentration or when the dose–response profile did not satisfy the monotonic criterion, the result was reported as not determined (n.d.; MIC > 25 mg/mL).

### 4.9. Cytotoxicity Assay (CCK-8)

The cytotoxic activity of *I. helenium* root extracts was evaluated using the Cell Counting Kit-8 (CCK-8) assay according to the manufacturer’s instructions. SK-MEL-28 melanoma cells were used as a cancer model, while HEK293 cells were included as a non-cancerous control to evaluate the selectivity of the extracts and their potential cytotoxic effects on non-cancerous cells. Cell viability was verified by trypan blue exclusion (>90% viable cells) prior to seeding.

*Cell lines and culture conditions*. SK-MEL-28 and HEK293 cells were cultured in Dulbecco’s Modified Eagle Medium (DMEM) supplemented with 10% (*v*/*v*) fetal bovine serum (FBS) and 1% (*v*/*v*) penicillin–streptomycin solution. Cells were maintained at 37 °C in a humidified atmosphere containing 5% CO_2_. The culture medium was replaced every 2–3 days. Upon reaching approximately 80–90% confluence, cells were washed with phosphate-buffered saline (PBS) and detached using 0.25% trypsin–0.1% EDTA solution (1 min at 37 °C). Cell viability was assessed using the trypan blue exclusion assay and a Neubauer hemocytometer. Only cell suspensions exhibiting a viability greater than 90% were used for subsequent experiments.

*Extract preparation*. Dried *I. helenium* root extracts obtained using *n*-hexane, ethyl acetate, and 70% ethanol were reconstituted in DMSO to prepare stock solutions (10 mg/mL). Working solutions were prepared by serial dilution in DMEM to final concentrations in the wells of 0.005, 0.010, and 0.020 mg/mL. The final concentration of DMSO in the culture medium did not exceed 0.2% (*v*/*v*). Vehicle control wells contained the same final concentration of DMSO as the extract-treated wells. This approach was used to ensure that any observed differences in cell viability were attributable to the extracts rather than to solvent-related effects.

*Experimental design*. For experimental treatments, cells were divided into the following groups: (i) negative control (DMEM), (ii) vehicle control (0.2% DMSO), (iii) positive control (20 µM cisplatin), and cells treated with different *I. helenium* root extracts. Background wells containing culture medium and CCK-8 reagent without cells were included for background correction.

*Assay procedure.* SK-MEL-28 and HEK293 cells were seeded into 96-well plates at a density of 12,000 cells per well in 100 µL of culture medium. Plates were incubated at 37 °C in a humidified atmosphere containing 5% CO_2_ for 24 h to allow cell attachment. After incubation, the medium was removed and replaced with fresh medium containing the respective treatments, followed by incubation for a further 24 h at 37 °C in 5% CO_2_. Following the 24 h exposure period, 10 µL of CCK-8 reagent was added to each well, and plates were incubated for an additional 1 h at 37 °C. The absorbance was measured at 450 nm. Each experiment included three biological replicates, each analyzed in triplicate technical replicates.

*Data analysis.* Cell viability (%) was calculated relative to the DMEM negative control after correction for the corresponding background wells. IC_50_ values were estimated from the observed concentration–viability profiles. When cell viability crossed the 50% threshold within the tested concentration range, IC_50_ values were obtained by linear interpolation between the two concentrations immediately surrounding the 50% viability level. Only three concentrations were tested, formal dose–response curve fitting was not performed and the resulting IC_50_ values should be regarded as approximate estimates. When the IC_50_ exceeded the highest tested concentration (0.020 mg/mL), it was reported as > 0.020 mg/mL. In such cases, exact IC_50_ values could not be determined within the tested concentration range and any derived ratios should be interpreted as lower-bound estimates only. The selectivity index (SI) was calculated as the ratio of the HEK293 IC_50_ to the SK-MEL-28 IC_50_. When either IC_50_ exceeded 0.020 mg/mL, the corresponding SI values were expressed as lower-bound estimates.

### 4.10. Statistical Analysis

Raw laboratory data were compiled, and initial calculations (means, standard deviations, calibration curves) were performed in Microsoft Excel 2021 (Microsoft Corporation, Redmond, WA, USA). All statistical analyses were performed using Python (version 3.13.13) with the SciPy (v1.17.1), scikit-learn (v1.6.1), pandas (v2.2.3), NumPy (v2.3.5), Matplotlib (v3.10.8), and Seaborn (v0.13.2) libraries.

Given the small sample size and the pronounced heterogeneity of variance between groups, non-parametric descriptive statistics were used throughout, and formal Shapiro–Wilk and Levene tests were therefore not applied. For the phytochemical, antioxidant, and cytotoxicity analyses, three independent biological replicates were included per origin–solvent combination, with each replicate measured in technical triplicate. For the antimicrobial assay, each extract–microorganism combination was evaluated in three technical replicate wells within a single independent experiment (*n* = 3 technical replicates per condition). Data are reported as median (Q1–Q3), except for cell viability values, which are additionally reported as mean ± SD to allow direct comparison with the reference control (DMEM = 100%). Global effects of extraction solvent and raw material origin on TPC and on the four antioxidant assays (FRAP, DPPH, ABTS, CUPRAC) were assessed by the Kruskal–Wallis test (*df* = 2) applied separately to each factor. Effect size was estimated by epsilon-squared (*ε*^2^), with *ε*^2^ > 0.26 interpreted as a large effect. When the Kruskal–Wallis test indicated a significant global effect, Dunn’s post hoc test with Bonferroni correction for multiple comparisons was used to identify pairwise differences. The same non-parametric strategy (Kruskal–Wallis followed by Dunn’s post hoc) was applied both to the three-solvent global comparison and to the nine origin–solvent group comparison. For the antimicrobial activity data, the effect of extraction solvent (*n*-hexane vs. 70% ethanol) on each microbial strain was tested by the two-tailed Mann–Whitney *U* test. For the cytotoxicity data, the difference in cell viability between HEK293 and SK-MEL-28 cells at each extract concentration was tested by the two-tailed Mann–Whitney *U* test. Suspected outlier values were identified by the combined application of the Grubbs test and Dixon’s *Q* test (both at *α* = 0.05). Only values confirmed by both tests were excluded from further statistical analysis, as documented in the corresponding Results subsection. The full UV–Vis absorbance spectra (200–800 nm, 1 nm resolution) of the nine extracts, as well as the integrated bioactivity matrix, were independently standardized by *z*-score transformation (mean = 0, standard deviation = 1) prior to analysis to compensate for the different measurement scales. *K. pneumoniae* inhibition data were excluded from the integrated matrix because growth inhibition remained below 20% across all evaluated *n*-hexane and 70% ethanol extracts, and neither the MIC_50_ nor the MIC_90_ threshold was reached. Principal component analysis (PCA) was applied to visualize sample grouping in PC1–PC2 space and to identify the main directions of variance. Hierarchical cluster analysis (HCA) was performed using Ward linkage on Euclidean distances of the standardized data; both samples and variables were independently clustered, and the resulting dendrograms were used to reorder the rows and columns of the heatmap. Pairwise Spearman rank correlation coefficients (*ρ*) were computed among all bioactivity variables using the median values of the nine origin–solvent groups; raw *p*-values were reported without correction for multiple comparisons. Results with *p* < 0.05 were considered statistically significant, and significance levels are indicated throughout the manuscript as * *p* < 0.05, ** *p* < 0.01, and *** *p* < 0.001. Artificial intelligence tools were used for language editing, figure formatting, and assisting with Python code development. All AI-generated outputs were reviewed and verified by the authors.

## 5. Conclusions

The present study suggests that, under the experimental conditions examined, the choice of extraction solvent is an important determinant of the phytochemical and biological profiles of *Inula helenium* root extracts, yielding two functionally distinct bioactive fractions with clearly distinguishable bioactivity profiles. These findings suggest that *I. helenium* preparations may be tailored to specific applications through targeted solvent selection and support the consideration of different solvent extracts of the species as complementary bioactive matrices arising from a common starting material, rather than as a single, uniform phytotherapeutic preparation.

The present data also suggest that, in unfractionated hydroalcoholic *I. helenium* root extracts, activity against the tested Gram-positive pathogen (*S. aureus*) and yeast (*C. albicans*) may be more closely associated with the polar polyphenol-rich fraction than with the lipophilic fraction traditionally regarded as the principal antimicrobial component of the species, supporting a broader multi-constituent, polarity-dependent bioactivity framework. Conversely, within the systematic origin-by-solvent design employed here, the low-polarity *n*-hexane and ethyl acetate extracts produced a preferential cytotoxic response toward SK-MEL-28 melanoma cells relative to HEK293 reference cells, with the cultivated Botanical Garden *n*-hexane extract exhibiting the strongest response among the tested extracts. The limited activity observed against the tested *K. pneumoniae* strain is consistent with the well-recognized resistance architecture of Gram-negative pathogens. It suggests that, at the concentrations evaluated in the present study, *I. helenium* extracts are unlikely to be effective as stand-alone antimicrobials against such organisms.

Although these findings require confirmation in larger, independently replicated studies, they provide an initial framework for the rational selection of extraction solvents based on the intended biological activity. Beyond their descriptive value, the results tentatively suggest two broader implications: (i) that a single medicinal plant material may, through solvent selection alone, be preferentially enriched for either antioxidant and antimicrobial or melanoma-targeted cytotoxic activities, potentially contributing to more rational standardisation of phytotherapeutic preparations; and (ii) that whole *I. helenium* extracts may provide a complementary bioactivity profile to that of isolated sesquiterpene lactones, potentially through additive or synergistic contributions of co-extracted constituents. Future studies should prioritise chromatographic identification and quantification of the sesquiterpene lactones and phenolic constituents underlying the observed biological activities, bioassay-guided fractionation of the low-polarity extracts exhibiting preferential cytotoxicity toward melanoma cells, expansion of cytotoxicity testing to a broader panel of malignant and non-malignant cell lines, in vivo evaluation of the most promising *n*-hexane fractions, and independent replication of the antimicrobial findings using appropriate reference antimicrobial controls and complementary non-optical viability assays. Expansion of the sample set across multiple populations, growing seasons, and cultivation conditions would further strengthen the multivariate patterns identified in this study and facilitate the development of more robustly standardised phytotherapeutic preparations.

## Figures and Tables

**Table 1:** Total phenolic content (TPC) and antioxidant activity of *Inula helenium* L. root extracts obtained with three solvents of different polarities from three distinct raw material sources.

Origin	Solvent	TPC ^a^	FRAP ^b^	DPPH ^b^	ABTS ^b^	CUPRAC ^b^
	*n*-hexane	7.27 (7.09–7.47) ^B^	18.96 (18.44–19.48) ^B^	1.88 (1.60–2.15) ^B^	18.96 (18.73–19.19) ^B^	91.47 (85.82–97.12) ^B^
Pharmacy	ethyl acetate	12.61 (12.11–13.12) ^B^	26.51 (25.97–27.04) ^B^	4.08 (4.00–4.46) ^B^	16.46 (16.39–16.52) ^B^	29.24 (29.17–29.30) ^B^
	70% ethanol	13.93 (13.73–14.12) ^A^	174.06 (174.02–174.10) ^A^	136.24 (133.86–138.38) ^A^	162.92 (160.84–165.00) ^A^	451.76 (446.05–457.47) ^A^
	*n*-hexane	8.25 (7.76–8.75) ^B^	28.31 (27.95–28.67) ^B^	7.99 (7.85–8.95) ^B^	26.21 (25.76–26.66) ^B^	79.88 (78.24–81.53) ^B^
Wild	ethyl acetate	13.37 (13.23–13.51) ^B^	42.00 (41.88–42.12) ^B,^*	5.10 (4.98–5.39) ^B^	45.52 (45.49–45.55) ^B^	126.11 (120.35–132.72) ^B^
	70% ethanol	38.59 (38.49–38.68) ^A,^*	98.83 (98.77–98.90) ^A^	177.29 (174.28–178.76) ^A,^*	328.93 (327.95–329.91) ^A,^*	541.62 (537.68–545.55) ^A,^*
	*n*-hexane	8.50 (7.98–9.01) ^B^	20.54 (20.53–20.55) ^B^	3.98 (3.71–4.08) ^B^	7.78 (7.24–8.33) ^B^	50.24 (50.13–50.34) ^B^
Botanical Garden	ethyl acetate	10.81 (10.43–11.19) ^B^	17.72 (17.52–17.93) ^B^	1.88 (1.87–2.19) ^B^	7.05 (5.75–8.36) ^B,^*	30.12 (28.90–30.94) ^B,^*
	70% ethanol	9.02 (8.93–9.10) ^A^	126.32 (125.38–127.26) ^A^	94.00 (92.17–98.17) ^A^	125.00 (124.83–125.17) ^A^	278.68 (276.60–280.76) ^A^
Kruskal–Wallis ^c^						
Solvent effect		*H* = 11.10	*H* = 11.47	*H* = 17.10	*H* = 11.37	*H* = 12.30
		*p* = 0.004	*p* = 0.003	*p* < 0.001	*p* = 0.003	*p* = 0.002
		*ε*^2^ = 0.58	*ε*^2^ = 0.63	*ε*^2^ = 0.66	*ε*^2^ = 0.63	*ε*^2^ = 0.61
Origin effect		*H* = 1.84	*H* = 1.56	*H* = 5.10	*H* = 4.68	*H* = 3.66
		*p* = 0.399	*p* = 0.459	*p* = 0.078	*p* = 0.096	*p* = 0.160

^a^ Total phenolic content expressed as milligrams of gallic acid equivalents per gram of dry extract (mg GAE/g DE). ^b^ Antioxidant activity expressed as micromoles of Trolox equivalents per gram of dry extract (µmol TE/g DE). ^c^ Kruskal–Wallis non-parametric test (*df* = 2) was used as the global comparison; *ε*^2^—epsilon-squared effect size; ns—not significant (*p* > 0.05). ^A,B^ indicate statistically significant differences between solvents within each assay (pooled across origins; *p* < 0.05): groups sharing the same letter do not differ significantly, while groups with different letters do. The asterisk (*) marks individual origin–solvent groups that participated in the only pairwise origin–solvent contrasts reaching significance after Bonferroni correction across the nine groups. Abbreviations: DE—dry extract; GAE—gallic acid equivalents; TE—Trolox equivalents.

**Table 2:** Absorbance maxima (*λ*_max_) and area-under-curve (AUC) values for UV–Vis spectra of *Inula helenium* L. root extracts (200–450 nm).

Origin	Solvent	λ_max_ 1 (nm)	A_1_	λ_max_ 2 (nm)	A_2_	AUC (±SD)	AUC Range (nm)
Pharmacy	*n*-hexane	211	0.898	–	–	18.77 ± 0.18	200–350
Wild		211	0.855	–	–	19.95 ± 0.23	200–350
Botanical Garden		211	0.843	–	–	18.56 ± 0.11	200–350
Pharmacy	ethyl acetate	235	0.694	251	0.573	45.68 ± 0.31	200–450
Wild		234	0.679	252	0.596	44.71 ± 0.17	200–450
Botanical Garden		234	0.675	252	0.598	44.85 ± 0.22	200–450
Pharmacy	70% ethanol	203	2.773	327	0.637	142.77 ± 0.25	200–450
Wild		203	1.627	326	0.815	116.81 ± 0.13	200–450
Botanical Garden		203	2.584	326	0.638	128.18 ± 0.28	200–450

*λ*_max_ denotes the wavelength of maximum absorbance identified using a peak prominence threshold of ≥0.03 AU; A represents the absorbance value at the corresponding *λ*_max_, and AUC indicates the area under the absorbance curve calculated using the trapezoidal method. The absorbance range of 200–350 nm was used for *n*-hexane extracts because no significant absorbance was observed above 350 nm, whereas the 200–450 nm range was used for ethyl acetate and 70% ethanol extracts.

**Table 3:** Minimum inhibitory concentrations (MIC_50_, MIC_90_) of *Inula helenium* L. root extracts against *Staphylococcus aureus*, *Klebsiella pneumoniae*, and *Candida albicans*.

Origin	Solvent	*S. aureus*		*K. pneumoniae*		*C. albicans*	
		MIC_50_ (mg/mL)	MIC_90_ (mg/mL)	MIC_50_ (mg/mL)	MIC_90_ (mg/mL)	MIC_50_ (mg/mL)	MIC_90_ (mg/mL)
Pharmacy	*n*-hexane	n.d.	n.d.	n.d.	n.d.	n.d.	n.d.
	70% ethanol	12.5	25	n.d.	n.d.	12.5	12.5
Wild	*n*-hexane	25	n.d.	n.d.	n.d.	n.d.	n.d.
	70% ethanol	25	25	n.d.	n.d.	3.125	25
Botanical Garden	*n*-hexane	n.d.	n.d.	n.d.	n.d.	n.d.	n.d.
	70% ethanol	12.5	12.5	n.d.	n.d.	3.125	6.25
Solvent effect at 25 mg/mL ^a^		*p* < 0.001 ***		*p* = 0.065 (ns)		*p* < 0.001 ***	

MIC_50_ and MIC_90_ were defined as the lowest tested concentration at which the median growth inhibition reached ≥50% and ≥90%, respectively, and at which all higher tested concentrations also met the same threshold (monotonic dose–response criterion; see Section 4.8). Values are derived from *n* = 3 technical replicate wells per condition within a single independent experiment across eight extract concentrations ranging from 0.195 to 25 mg/mL; full dose–response profiles are provided in Figure 5 and Table S1. Individual per-well inhibition values were capped at the [0, 100] interval prior to analysis. n.d. = not determined (threshold not reached, or dose–response profile did not satisfy the monotonic criterion; MIC > 25 mg/mL). Superscript ^a^ Mann–Whitney *U* test comparing *n*-hexane and 70% ethanol extracts at 25 mg/mL, pooled across raw material origins; *** *p* < 0.001; ns = not significant. Raw material origin had no significant effect on growth inhibition at 25 mg/mL for any microorganism (Kruskal–Wallis, *p* > 0.58). Ethyl acetate extracts were excluded due to optical interference (see Section 2.5).

**Table 4:** Cytotoxic activity of *Inula helenium* L. root extracts against HEK293 and SK-MEL-28 cell lines.

Origin	Solvent	Conc. (mg/mL)	HEK293 Viability (%)	SK-MEL-28 Viability (%)	Δ*V* (HEK − SK)	*p* Value ^a^	Sig.
Pharmacy		0.005	87.5 ± 8.3	83.7 ± 11.9	+3.8	0.529	ns
	*n*-hexane	0.010	82.1 ± 7.7	89.9 ± 8.5	−7.9	0.071	ns
		**0.020**	**67.6 ± 6.5**	**47.8 ± 2.3**	**+19.8**	**0.0004**	***
		0.005	80.6 ± 2.4	86.9 ± 8.2	−6.3	0.128	ns
	ethyl acetate	0.010	92.7 ± 2.8	86.3 ± 7.4	+6.4	0.142	ns
		**0.020**	**71.6 ± 5.1**	**62.7 ± 2.6**	**+8.9**	**0.0003**	***
		0.005	108.8 ± 5.0	108.7 ± 14.0	+0.0	0.779	ns
	70% ethanol	0.010	104.2 ± 13.7	104.6 ± 6.8	−0.3	0.445	ns
		0.020	98.6 ± 8.4	99.1 ± 16.6	−0.5	0.950	ns
Wild		0.005	94.8 ± 6.1	103.2 ± 9.2	−8.4	0.040	*
	*n*-hexane	0.010	77.3 ± 7.9	81.8 ± 9.0	−4.5	0.232	ns
		**0.020**	**73.8 ± 3.8**	**58.6 ± 3.7**	**+15.2**	**0.0003**	***
		0.005	81.5 ± 6.7	103.4 ± 6.1	−21.9	0.001	**
	ethyl acetate	0.010	80.4 ± 8.5	87.1 ± 9.1	−6.7	0.181	ns
		**0.020**	**67.3 ± 2.9**	**49.5 ± 3.1**	**+17.8**	**0.0007**	***
		0.005	100.6 ± 3.3	98.2 ± 4.2	+2.4	0.181	ns
	70% ethanol	0.010	97.6 ± 5.3	109.5 ± 6.5	−11.9	0.011	*
		0.020	108.5 ± 3.3	104.9 ± 3.7	+3.6	0.181	ns
		0.005	100.6 ± 18.7	93.0 ± 21.3	+7.6	0.710	ns
	*n*-hexane	0.010	84.5 ± 12.9	79.9 ± 27.3	+4.6	0.945	ns
		**0.020**	**52.9 ± 3.2**	**35.3 ± 2.5**	**+17.5**	**0.0003**	***
		0.005	87.9 ± 6.4	94.8 ± 28.4	−6.9	0.755	ns
Botanical Garden	ethyl acetate	0.010	83.1 ± 8.5	84.4 ± 17.2	−1.3	0.628	ns
		**0.020**	**71.3 ± 3.8**	**57.6 ± 2.7**	**+13.8**	**0.0012**	**
		0.005	92.0 ± 5.3	117.3 ± 18.7	−25.3	0.053	ns
	70% ethanol	0.010	105.6 ± 7.7	113.0 ± 10.6	−7.4	0.174	ns
		0.020	96.8 ± 5.4	101.4 ± 19.9	−4.5	0.710	ns

Values are expressed as mean ± SD of *n* = 3 biological replicates, each measured in technical triplicate. Δ*V* = mean difference (HEK293 − SK-MEL-28); positive values indicate greater cytotoxicity toward SK-MEL-28 (cancer cells). Cell viability was calculated relative to the DMEM negative control (set as 100%). ^a^ Two-tailed Mann–Whitney *U* test comparing HEK293 vs. SK-MEL-28 viability at each concentration. Significance levels: *** *p* < 0.001; ** *p* < 0.01; * *p* < 0.05; ns = not significant. Six extracts showed statistically significant preferential cytotoxicity toward SK-MEL-28 cells relative to HEK293 cells at 0.020 mg/mL (highlighted with bold rows and **/*** markers in Δ*V*/*p* columns).

**Table 5:** Lower-bound estimates of selectivity indices of *Inula helenium* L. root extracts based on the highest tested concentration (0.020 mg/mL).

Origin	Solvent	HEK293 IC_50_	SK-MEL-28 IC_50_	SI ^b^	Rank
Botanical Garden	*n*-hexane	>0.020 mg/mL	0.017 mg/mL	>1.50	1
Pharmacy	*n*-hexane	>0.020 mg/mL	0.020 mg/mL	>1.41	2
Wild	ethyl acetate	>0.020 mg/mL	0.020 mg/mL	>1.36	3
Wild	*n*-hexane	>0.020 mg/mL	>0.020 mg/mL	>1.26	4
Botanical Garden	ethyl acetate	>0.020 mg/mL	>0.020 mg/mL	>1.24	5
Pharmacy	ethyl acetate	>0.020 mg/mL	>0.020 mg/mL	>1.14	6
Pharmacy	70% ethanol	>0.020 mg/mL	>0.020 mg/mL	N/A (non-toxic)	–
Wild	70% ethanol	>0.020 mg/mL	>0.020 mg/mL	N/A (non-toxic)	–
Botanical Garden	70% ethanol	>0.020 mg/mL	>0.020 mg/mL	N/A (non-toxic)	–

^b^ Selectivity Index (SI) = HEK293 IC_50_/SK-MEL-28 IC_50_. As HEK293 viability did not reach 50% within the tested concentration range, exact HEK293 IC_50_ values could not be determined. Therefore, the reported SI values represent lower-bound estimates only. Higher SI values indicate a greater difference between HEK293 and SK-MEL-28 cytotoxic responses within the investigated concentration range. SK-MEL-28 IC_50_ values falling within the tested concentration range were estimated by linear interpolation between the two concentrations surrounding the 50% viability threshold.

## Data Availability

The original contributions presented in this study are included in the article/Supplementary Materials. Further inquiries can be directed to the corresponding author.

## Supplementary Materials

The following supporting information can be downloaded at https://www.mdpi.com/article/10.3390/molecules31173019/s1. Table S1: Full dose–response profiles for the antimicrobial activity of *Inula helenium* L. root extracts against *Staphylococcus aureus*, *Klebsiella pneumoniae*, and *Candida albicans*.

## Abbreviations

The following abbreviations are used in this manuscript:

ABTS	2,2′-Azino-bis(3-ethylbenzothiazoline-6-sulfonic acid)
ATCC	American Type Culture Collection
AUC	Area under the curve
BHI	Brain Heart Infusion
CCK-8	Cell Counting Kit-8
CUPRAC	Cupric ion reducing antioxidant capacity
DE	Dry extract
DMEM	Dulbecco’s Modified Eagle Medium
DMSO	Dimethyl sulfoxide
DPBS	Dulbecco’s phosphate-buffered saline
DPPH	2,2-Diphenyl-1-picrylhydrazyl
EDTA	Ethylenediaminetetraacetic acid
EtOAc	Ethyl acetate
EtOH	Ethanol
FBS	Fetal bovine serum
FRAP	Ferric reducing antioxidant power
GAE	Gallic acid equivalent
HCA	Hierarchical cluster analysis
HEK293	Human embryonic kidney 293 cell line
IC_50_	Half-maximal inhibitory concentration
IQR	Interquartile range
MIC	Minimum inhibitory concentration
MIC_50_	Minimum inhibitory concentration inhibiting 50% of growth
MIC_90_	Minimum inhibitory concentration inhibiting 90% of growth
PCA	Principal component analysis
PC1, PC2	First and second principal components
P/S	Penicillin/streptomycin
Q1–Q3	First to third quartile (interquartile range)
SD	Standard deviation
SI	Selectivity index
SK-MEL-28	Human melanoma cell line
TE	Trolox equivalent
TPC	Total phenolic content
TPTZ	2,4,6-Tri(2-pyridyl)-s-triazine
Trolox	6-Hydroxy-2,5,7,8-tetramethylchroman-2-carboxylic acid
UAE	Ultrasound-assisted extraction
UV–Vis	Ultraviolet–visible (spectroscopy)
*λ*_max_	Wavelength of maximum absorbance
ε^2^	Epsilon-squared (effect size)
*ρ*	Spearman’s rank correlation coefficient
