# Supplementary material for: Solvent-Dependent Phytochemical Profiles, Antioxidant and Antimicrobial Bioactivities, and Melanoma-Selective Cytotoxicity of Inula helenium L. Root Extracts
Source: Molecules. 2026 Aug 28;31(17):3019. doi: 10.3390/molecules31173019 (PMC13567029; doi:10.3390/molecules31173019)
Supplement: Supplementary file 1 [file molecules-31-03019-s001.zip › molecules-4522645-supplementary.pdf]

Table S1. Full dose–response profiles for the antimicrobial activity of *Inula helenium* L. root extracts against *Staphylococcus aureus*, *Klebsiella pneumoniae*, and *Candida albicans*. Median growth inhibition (%) at all eight tested extract concentrations (0.195–25 mg/mL) for the three raw material origins and two extraction solvents.

Microorganism	Origin	Solvent	Median growth inhibition (%) at concentration (mg/mL)								MIC_{50} (mg/mL)	MIC_{90} (mg/mL)
			25	12.5	6.25	3.125	1.563	0.781	0.391	0.195		
S. aureus	Pharmacy	*n*-hexane	46.6	6.8	0.0	0.0	0.0	0.0	0.0	0.0	n.d.	n.d.
S. aureus	Pharmacy	70% ethanol	100.0	87.5	0.0	0.0	0.0	0.0	0.0	0.0	12.5	25
S. aureus	Wild	*n*-hexane	63.4	13.3	0.0	0.0	0.0	0.0	0.0	0.0	25	n.d.
S. aureus	Wild	70% ethanol	96.5	13.0	0.0	0.0	0.0	0.0	0.0	0.0	25	25
S. aureus	Botanical Garden	*n*-hexane	35.5	0.0	0.0	0.0	0.0	0.0	0.0	0.0	n.d.	n.d.
S. aureus	Botanical Garden	70% ethanol	100.0	100.0	0.0	0.0	0.0	0.0	0.0	0.0	12.5	12.5
K. pneumoniae	Pharmacy	*n*-hexane	13.2	7.1	12.7	0.0	0.0	0.0	0.0	5.1	n.d.	n.d.
K. pneumoniae	Pharmacy	70% ethanol	9.6	7.8	10.9	0.0	0.0	0.0	4.5	3.4	n.d.	n.d.
K. pneumoniae	Wild	*n*-hexane	11.4	5.6	10.3	0.0	0.0	0.0	10.4	8.8	n.d.	n.d.
K. pneumoniae	Wild	70% ethanol	0.0	0.0	0.5	0.0	0.0	0.0	0.0	5.4	n.d.	n.d.
K. pneumoniae	Botanical Garden	*n*-hexane	4.0	10.3	17.5	4.1	6.5	3.8	7.1	3.3	n.d.	n.d.
K. pneumoniae	Botanical Garden	70% ethanol	18.3	4.7	7.9	0.0	0.0	0.3	8.3	4.5	n.d.	n.d.
C. albicans	Pharmacy	*n*-hexane	18.8	42.1	46.7	50.8	30.1	31.3	33.1	42.8	n.d.	n.d.
C. albicans	Pharmacy	70% ethanol	100.0	100.0	0.0	0.0	0.0	5.7	15.2	3.8	12.5	12.5
C. albicans	Wild	*n*-hexane	23.9	0.0	0.0	0.0	0.0	0.0	0.0	12.4	n.d.	n.d.
C. albicans	Wild	70% ethanol	100.0	81.5	60.2	56.4	25.4	17.5	22.5	36.3	3.125	25
C. albicans	Botanical Garden	*n*-hexane	46.4	35.7	26.9	29.3	8.8	10.5	3.7	5.7	n.d.	n.d.
C. albicans	Botanical Garden	70% ethanol	100.0	100.0	100.0	70.3	41.9	31.2	29.9	41.0	3.125	6.25

Values are medians of $n = 3$ replicates per group. Individual per-well inhibition values were capped at the [0, 100] interval prior to computing medians; values of 0.0 indicate that measured absorbance was equal to or greater than the corresponding DMSO control (no growth inhibition), and values of 100.0 indicate complete growth inhibition (raw inhibition ≥ 100% capped to the physiological maximum). MIC_{50} and MIC_{90} were defined as the lowest tested concentration at which the median growth inhibition reached ≥ 50% and ≥ 90%, respectively, and at which all higher tested concentrations also met the same threshold (monotonic dose–response criterion). n.d. = not determined. Ethyl acetate extracts were excluded due to optical interference (see Section 2.5).
